# An Effective Approach for Recognition of Crop Diseases Using Advanced Image Processing and YOLOv8

**DOI:** 10.1002/fsn3.71504

**Published:** 2026-02-09

**Authors:** Muhammad Nouman Noor, Muhammad Masab, Farah Haneef, Muzammil Hussain, Mateen Yaqoob, Tehseen Mazhar, Muhammad Amir Khan, Ghadah Aldehim

**Affiliations:** ^1^ Department of AI and Data Science National University of Computer and Emerging Sciences (FAST‐NUCES) Islamabad Pakistan; ^2^ Department of Computer Science HITEC University Taxila Pakistan; ^3^ Department of Software Engineering Capital University of Science and Technology Islamabad Pakistan; ^4^ School of Computer Science National College of Business Administration and Economics Lahore Pakistan; ^5^ Department of Computer Science and Information Technology School Education Department, Government of Punjab Layyah Pakistan; ^6^ Faculty of Computer and Mathematical Sciences Universiti Teknologi MARA Shah Alam Selangor Malaysia; ^7^ Department of Information Systems, College of Computer and Information Sciences Princess Nourah bint Abdulrahman University Riyadh Saudi Arabia

**Keywords:** artificial intelligence, crop diseases, image processing, YOLOv8

## Abstract

The spread of plant diseases in important crops that influence the economy, particularly in Asia, such as tomatoes, coffee, cucumbers, olives, and wheat, poses a serious threat to agricultural production and global food security. Traditional detection methods are frequently labor‐intensive, slow, and lack the public availability of data, which subsequently impacts the model's generalizability and implementation in the real world for practical use. For this purpose, a computer‐aided approach is required to detect and classify diseases using crop images. In this research, images are initially processed using advanced image processing techniques like local contrast enhancement, wavelet transform, sigmoid correction, gamma correction, and median filtering, which are then evaluated using mean squared error and peak signal‐to‐noise ratio. After the processing phase, we utilize an advanced deep learning model, YOLOv8, to segment and classify crop diseases using publicly available data. This hybrid dataset includes data collection of 32 diseases. Using a large dataset, which comprises 32 diseases, to train our model, we implemented Transfer Learning using YOLOv8. We performed segmentation and classification with excellent recall and precision, with a recall of 0.94 and an overall accuracy of 92.567. The evaluation measures show dependable performance in crop disease identification across various circumstances. This will not only enhance the early disease detection in key crops but also reduce the intervention of experts, resulting in improved early disease diagnosis and the aversion of significant crop losses.

## Graphical abstract

The performance of processed images is evaluated using mean‐squared‐error and peak‐signal‐to‐noise ratio. After the processing phase, an advanced deep learning model, YOLOv8, was used for the segmentation and classification of crop diseases. Using a large dataset comprising 32 diseases to train our model, we implemented Transfer Learning using YOLOv8. We performed segmentation and classification with excellent recall and precision, with a recall of 0.94 and an overall accuracy of 92.567.

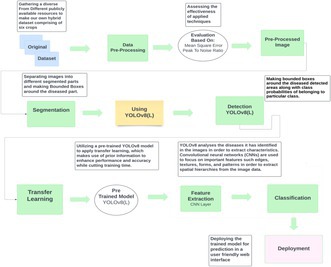

## 1 Introduction

Agriculture is the central research area worldwide nowadays (Iqbal et al. 2018). The production of crops plays a significant part in developing the country's economy (Agarwal et al. 2021). Food waste has grown to be an essential worldwide problem with serious negative effects on the economy, ecology, and society, considering just the economy. The Food and Agriculture Organization of the United Nations (FAO) estimates that 1.3 billion tons of food are lost or wasted annually globally, accounting for one‐third of all food production (Gustavsson et al. 2011). Early detection and prevention of plant diseases are essential in agricultural technology and strategy for commercial farms. Traditional manual visual observation techniques for diagnosing diseases are typically labor‐intensive, inefficient, and significantly raise overhead costs (Fu et al. 2020; SepúLveda et al. 2020). Furthermore, conventional machine learning techniques are not suitable for real‐world detection with limited data and situations with irregular and complicated backgrounds. Subsequently, in many cases, deep learning has shown exceptional results, particularly in the field of computer vision in this area (Wu et al. 2018; Lu et al. 2018; Li et al. 2018). The integration of deep learning in agriculture, specifically in disease detection and classification, shows promising results; for example, models such as YOLOv4 have been used to accurately identify plant diseases, which has significantly decreased crop losses (Aldakheel et al. 2024). In detecting diseases at early stages, deep learning techniques played an important part, which is essential for putting timely therapies into place and minimizing harm (Albattah et al. 2022; Sanida et al. 2022). Precision farming and other sustainable agricultural techniques significantly enhance food yield and security by improving dietary diversity and reducing micronutrient deficiencies (Nahar et al. 2024). Food insecurity has been made worse by the COVID‐19 pandemic, which has made already vulnerable populations more vulnerable by upsetting supply networks and restricting market access (Adelaja and George 2021). Initiatives to reduce food waste may significantly impact the environment and food security. For instance, if food waste were reduced by just 25% worldwide, 870 million people might be fed, significantly reducing the number of hungry people. Furthermore, reducing food waste would decrease greenhouse gas emissions, as food waste makes up around 8%–10% of global emissions (Liu et al. 2023). To create a more secure and sustainable food system, it is imperative to address food loss and waste challenges that could impact life worldwide (Varzakas and Smaoui 2024).

Convolutional Neural Networks (CNNs) have shown exceptional results, which has led to their being widely applied to image analysis applications, such as agriculture disease diagnosis. A study used a CNN‐based method to identify and categorize tomato crop illnesses. A deep learning model consisting of two convolutional layers and then pooling layers was used in the suggested approach. Their approach includes a deep learning model and follows the pattern of two convolutional layers and pooling layers. This model outperformed pre‐trained models like InceptionV3, ResNet152, and VGG19, with a high training accuracy of 98% and testing accuracy of 88.17%. CNNs' layers' ability to automatically learn and extract the features or pattern information from images makes them more suitable for classification tasks in agriculture (Sakkarvarthi et al. 2022). Subsequently, another study by Alnaggar et al. (2022) investigated the application of Vision Transformers in a different 2023 study, combining them with convolutional layers to diagnose rice crop diseases. This hybrid model benefited from ViTs' self‐attention mechanism, which focused on critical areas of the image to increase accuracy and provide a more thorough analysis. With CNNs being particularly good at feature extraction and ViTs providing better contextual comprehension while achieving an accuracy of 91.5%, both approaches have a lot of potential to be useful weapons in the battle against agricultural diseases (Alnaggar et al. 2022). Although research is going on in the area, a lot of improvements are still required in terms of performance. Therefore, this research is conducted to improve the recognition performance for crop disease. The major contributions of this research are as follows:

- Crop images are processed using advanced techniques like local contrast enhancement, wavelet transform, sigmoid correction, gamma correction, and median filtering, which are then evaluated using mean squared error and peak signal‐to‐noise ratio.
- The performance of processed images is evaluated using mean‐squared‐error and peak‐signal‐to‐noise ratio.
- After the processing phase, an advanced deep learning model, YOLOv8, was used for the segmentation and classification of crop diseases on publicly available data, a hybrid dataset including data collection of 32 diseases. Using a large dataset comprising 32 diseases to train our model, we implemented Transfer Learning using YOLOv8. We performed segmentation and classification with excellent recall and precision, with a recall of 0.94 and an overall accuracy of 92.567.

Section 2 has organized and presented the work of relevant studies; then, Section 3 effectively presents the methodology utilized for the study; afterwards, Section 4 explains the results achieved on the given method for segmentation and classification. Finally, the conclusion presents the research conclusion.

## 2 Literature Review

Agriculture is a critical domain affecting global food security and economic stability. It shows how important it is to detect disease in a timely and early stage for yield optimization and to create a practical and efficient method for identifying diseases, overcoming the limitations of conventional methods. For creating a more generalized and acceptable disease classification, they used a dataset containing eight samples subdivided into four levels across particular plant species (i.e., healthy, powdery mildew, black rot, and downy mildew). The research presents an innovative approach for plant disease identification using one class classifier with image segmentation Local Binary Patterns (LBPs) and conflict resolution. The Grab‐Cut algorithm ensures precise separation of leaf regions from complicated backgrounds. At the same time, the image acquisition enhances its practicality. Further, the LBPs transform images into integer‐valued matrices, capturing fine structural details crucial for disease identification. The core of their approach is to utilize One Class Classification using Support Vector Machines (SVMs) to identify outliers in health conditions, with conflict resolution strategies enhancing accuracy where symptoms might overlap and scoring a remarkable 95% accuracy in disease recognition. The model showcases notable generalization capabilities, successfully identifying diseases across various plant species, surpassing the constraints of a limited training dataset. However, the validation data size is still a concern and may result in overfitting. The study tackles the difficulties associated with identifying plant diseases. It provides new avenues for growing datasets, improving dispute settlement processes, and investigating precision agriculture uses for Allen compassing crop management (Pantazi et al. 2019). This research addresses the pressing issue of disease detection in crops, particularly targeting rice and corn, and emphasizes the importance and need for sustainable agricultural yield in a country heavily reliant on farming and points out that 47% of Pakistanis work in agriculture, which accounts for 18.9% of the nation's GDP. As the world's population rises, self‐sufficient techniques become essential to meet the growing demand for food production. The proposed solution uses pre‐trained models, including Inception V3 and ResNet 152, and adds a dense layer to achieve higher accuracies of 97.81% and 97.48% in corn crop disease detection. Notably, accuracy is improved by 82.20% and 99.10%, respectively, when the rice dataset is explicitly divided into major and minor disease categories. The outcomes outperform earlier techniques, demonstrating the effectiveness of their strategy. Anticipating the future, the paper suggests investigating diverse deep‐learning models and broadening the approach to include a range of plant diseases. This highlights the importance of the paper in the continuous progress of agricultural technology and disease control (Saeed et al. 2021).

The study by Thakur et al. (2021) introduces “PlantViT,” a transformer‐based automatic disease detection model that combines a CNN with a Vision Transformer. The goal is to create a deep learning method using Vision Transformers to detect plant ailments in leaf pictures. The model incorporates the capabilities of both the Vision Transformer and CNNs.

In the paper by Chouhan et al. (2019), we present an automated strategy to segment plant diseases from leaf images using Radial Basis Function Neural Networks (RBFNN). The photographs for this project were sourced from the IPM agriculture database repository. The experimental results show that the suggested RBFNN outperforms the other strategies in terms of segmentation accuracy.

The study by Patel et al. (2025) introduces a novel precision agriculture strategy by establishing a solid foundation for basil crop recognition using the YOLOv8 deep learning model. Basil poses unique monitoring challenges due to its various perspectives on different stages of growth and environmental conditions. To overcome these challenges and provide a comprehensive representation of the plant's diversity, a carefully curated library of basil photographs was produced.

To enable real‐time, autonomous agricultural decisions, Tariq et al. (2025) proposes an edge‐enabled smart agriculture architecture that blends IoT devices, rule‐based agentic AI, and lightweight deep learning. The system has two vision‐based models, one for crop recognition and one for weather classification, and is built on the MiT‐B0 Vision Transformer architecture for low‐resolution (128 × 128) image inputs.

Drones are used for crop monitoring, field surveying, pesticide management, spraying, mapping soil nutrient shortages, managing water stress, pre‐ and post‐harvest analysis, and other purposes. The utilization of drones allows for the speedy processing and evaluation of this data. As a result, this chapter contains a comprehensive review of the relevance and implications of drones (Chouhan et al. 2025). The study by Shukla et al. (2025) provides a thorough assessment of how cloud‐based platforms such as AgriCloud can transform farming by fostering more intelligent, effective, and sustainable practices. Robotic cutting point recognition in automated lychee harvesting presents significant challenges due to the unique geometric patterns of branches, leaves, and clustered fruits. Even minor positional errors might cause crop damage and operational difficulties (Wang et al. 2025).

The paper presents a novel crop leaf disease recognition method by utilizing an advanced architecture that combines deep learning and optimization techniques and addresses the challenge of accurate crop leaf disease recognition. The dataset comprises images of potato, cucumber, and tomato leaves affected by various diseases. The study addresses the problem of limited labeled training data and mitigates it with an effective data augmentation step using contrast enhancement and rotation operations. The suggested framework highlights the significance of each stage in the procedure and uses robust data augmentation to address dataset imbalances. The process includes adding more data, optimizing the DarkNet19 model, extracting deep features, and selecting features using the Improved Cuckoo Search algorithm. The selection of pre‐trained and fine‐tuning models, such as DarkNet‐19, reflects strategic decision‐making as a comparatively small number of parameters facilitates efficient training and feature extraction. Deep features are taken out of the global pooling layer, and the refined model is trained via transfer learning as part of the feature extraction process. The findings show notable increases in accuracy, with some disease classes seeing 100% improvement. The high accuracy levels demonstrate the potential utility of the suggested framework in identifying agricultural diseases. However, there is still improvement in exploring advanced and a wider range of CNN models, which lack a thorough analysis, offer more algorithmic specifics, and grow the dataset to make it more widely applicable. Despite its shortcomings, the work considerably improves crop disease detection systems by outperforming current methods and providing insightful information. Subsequent studies ought to investigate more complex CNN models, weigh the benefits and drawbacks of training on noisy images, and use a variety of datasets to thoroughly assess the framework's robustness (Thakur et al. 2021). In the vital domain of agriculture, where a significant portion of the global population relies on this sector, the detection and prevention of diseases in crops are paramount to ensure food security and economic stability. The study stresses the potentially disastrous effects of undiagnosed illnesses on output and financial stability. To overcome the difficulties presented by labor‐intensive, manual traditional approaches, the research focuses on the early detection of illnesses in different plant species. Leveraging a diverse dataset, the proposed framework detects 38 diseases across 14 plant species; the approach uses the technique of utilizing machine learning and transfer learning pre‐trained models like EfficientNetV2L, MobileNetV2, and ResNet152V2, which contribute effectively to the classification of diseases. The quantitative evaluation highlights its accuracy, precision, recall, and F1 score superiority. Interpretability is provided via the XAI framework, LIME, which increases the transparency of the model. The study also compares their approach with the previous technique, demonstrating its superiority in obtaining an accuracy of 99.63% with EfficientNetV2L. While the study lacks infrastructural limitations, it describes prospects, such as growing the dataset, improving pre‐processing methods, and adding geographic characteristics for sophisticated agricultural applications (Mehedi et al. 2022). The paper delves into the critical realm of agricultural sustainability, focusing on the problems caused by diseases that impact Pakistan's cotton crops, as cotton significantly contributes to the GDP. Identifying the significant economic impact of diseases like Cotton Leaf Curl Virus, Fusarium Wilt, and Bacterial Blight, the study implies deep learning techniques and genetic algorithms. The study uses a cotton leaves dataset infected with Fusarium wilt, Bacterial Blight, and Cotton Leaf Curl Virus, significantly reducing crop yields and threatening farmers' livelihoods. The study addresses the primary challenge of identifying diseases early and ensures the correct disease diagnosis while minimizing loss. The proposed solution includes a deep CNN with a transfer learning technique specifically utilizing the VGG‐16 model, eliminating the need for manual feature extraction while allowing the model to learn and recognize disease patterns automatically. The research compares different architectures, including Res‐Net and Inception; the comparative study shows the superiority of the proposed VGG‐16 model in terms of performance and accuracy; the model scored 98% accuracy. The study highlights the potentially useful applications of this research in actual agricultural practices by recommending the creation of an intuitive mobile application to assist cultivators in accurately identifying cotton leaf diseases (Naeem et al. 2023). The study uses an AI‐Hub dataset that includes photos of Korean crops with marked‐up metadata related to diseases. The dataset includes different crops, disease stages, and symptoms. The study addresses the critical challenge. By creating a comprehensive AI‐based solution, the research tackles the crucial problem of crop disease management. The image captioning model generates comprehensive diagnostic sentences using disease keywords from the National Crop Pest Management System and metadata from AI‐Hub. This entails assigning keywords to disease symptoms, creating sentences that diagnose diseases, and using text augmentation methods. The Image Captioning model uses a Transformer with default hyperparameters to extract features from InceptionV3. It uses BELU scores for quantitative evaluation in order to achieve high sentence generation performance, specifically in disease diagnosis; the mAP50 for the Object Detection model is 0.382, indicating a need for future development, the average BLEU score of the Image Captioning model is 64.96%, indicating strong performance in sentence production. The YOLOv5‐based Object Detection model is trained using datasets unique to the facility and outdoor crops. The solution consists of a comprehensive approach by making bounding boxes around affected areas, resulting in detailed diagnostic data, visual depiction and improved interpretability. The study offers a promising step toward technology driven crop management, with room for improvement, growth, and practical application. The integration of both textual and visual outputs enhances the solution's interpretability and usability and establishes the foundation for further developments in precision agriculture (Lee et al. 2023). The study addresses the critical need for automated disease detection in tomato plants using advanced deep learning techniques, as it poses a critical threat to the food supply and includes economic harm. The study targets the publicly accessible dataset consisting of 11,000 images of 10 different diseases of tomato plants. The paper proposed a multi‐stage method using deep learning, specifically Convolutional Neural Networks (CNNs) and pre‐trained models like DenseNet121, ResNet50V2, and Vision Transformer (VIT), to overcome the inherent difficulties of manual identification caused by intricate leaf designs and a variety of diseases and also it is significantly important to perform early detection, to overcome the crop losses. The process includes collecting datasets, pre‐processing the data to normalize and resize images, and using transfer learning to make use of pretrained models. The results show that DenseNet‐121 performs exceptionally well with the training accuracy of 99.88% and testing accuracy of 99.00%. DenseNet‐121 shows exceptional dominance, evaluation metrics results show its exceptional performance, which includes F1‐score, precision and recall. The study mainly focusses on the early diagnosis of diseases to make effective planting of tomato and emphasizes the use of deep learning techniques for that purposes to make agricultural practices more reliable and give handy information by comparing different deep learning techniques and their performances, also suggest ensemble technique for obtaining better performances and accuracies in detection of different diseases (Alzahrani and Alsaade 2023). Since agriculture is the main source of our food, effective disease management techniques must be used on crops. The study addresses the problem of limited labeled data availability while targeting a specific wheat crop, emphasizing the importance of labeled data for training, while comparing it with the traditional technique, which has a sparse and diverse dataset. This study addresses the problem of limited labeled data availability while focusing on the wheat crop; the study uses a pre‐trained model, Efficient Net, as a feature extractor and proposes a novel approach that includes a few short learning networks for the classification of wheat diseases that is effective in extracting pertinent data from a variety of datasets, including Plant Village and CGIAR Crop Diseases. The attention mechanism increases the computational efficiency and accuracy, increasing the model's usefulness. The model performs exceptionally well when accurately classifying wheat diseases; it achieved an astounding 93.19% accuracy on a manually compiled dataset of 18 wheat diseases from Google. The few‐shot learning method performs robustly against current techniques and successfully identifies diseases with minimum training data; nevertheless, a thorough comparison with state‐of‐the‐art methods would be beneficial. Although the suggested method is realistic and resource‐efficient, it has drawbacks. A thorough examination of scalability to manage a wider range of illnesses or crops is absent from the study. Furthermore, the manually gathered dataset's smaller size limits the evaluation's scope, which affects the comparison analysis's depth. The paper presents a few‐shot learning method for precision agriculture, addressing limited labeled data in crop disease classification, demonstrating its effectiveness, and highlighting future work for scalability (Alharbi et al. 2023). Deep crop offers a thorough method of predicting crop disease using different deep learning models, including CNN, VGG‐16, VGG‐19, and ResNet‐50. The study employs the “plant‐village” dataset, covering various crops comprising 10,000 images. The primary challenge addressed is the precise identification of plant diseases, also addressed the challenges of multi‐crop leaf disease detection. A multi‐phased strategy is used in the provided solution. The process flow includes data collection, pre‐processing model training using transfer learning (CNN, VGG16, VGG‐19, ResNet‐50), and evaluating the model using different metrics. Deep learning models, particularly ResNet‐50, significantly enhance hierarchical data representation in architectures, with evaluation metrics including accuracy, precision, recall, F1 score, training accuracy, training loss, validation accuracy, and validation loss demonstrate, Deep Crop achieves commendable accuracies across models: 98.60% (CNN), 92.39% (VGG‐16), 96.15% (VGG‐19), and 98.98% (ResNet‐50). The study not only attained superior accuracies but also emphasize the importance of the ResNet‐50 model for crop disease detection and chose to be integrated into a web application. The study addressed the problem of accurate disease identification and proposed a user‐friendly web application for remote diagnosis. The study contributes significantly to smart agriculture, offering a robust solution for timely disease identification and preventive measures. On the other hand, it makes suggestions for possible enhancements in thoroughly examining hyperparameters and verifying the actual implementation of the created web application. With its promise for improved crop health management and significant advancement in the use of AI in agriculture, Deep Crop is a key component of AI‐enabled agriculture (Islam et al. 2023). This research addresses the problem of identifying plant diseases on leaves, which is made more difficult by differences in leaf size, shape, and color. They utilize the YOLOv4 architecture, utilize the plant village dataset, and improve disease detection and classification accuracy. The model achieved an accuracy of 99.99%, demonstrating high precision and efficiency in recognizing multiple diseases and robustness against distortions such as noise, blurring, and lighting variations. The research also highlights certain drawbacks, which include the implementation of the model in different environments and reliance on annotated data quality and also emphasizes the further enhancement of images and expanding the dataset to prevent overfitting and for better generalizability (Aldakheel et al. 2024). The study uses an innovative approach, the ensemble Convolutional Neural Network (CNN) model, to offer an enhanced method for detecting diseases in tomato leaves. A comprehensive dataset for that purpose, which includes the nine distinct tomato leaf illnesses as well as healthy leaves, was used in the study. The study utilizes the four pre‐trained models (MobileNetV3Small, EfficientNetV2L, InceptionV3, and MobileNetV2) with two new CNN models that they presented. The study also utilizes particle swarm optimization to improve and fine‐tune that model and the grid search to optimize the weights. The ensemble models demonstrated an impressive 99.60% accuracy when tested with five‐fold cross‐validation. The study also identified certain drawbacks, such as the substantial processing power needed, and recommended further research to create lighter models for real‐time field deployment (Ulutaş and Aslantaş 2023). The study by Karthik et al. (2024) introduces a unique dual‐track architecture for crop disease and pest detection based on deep learning. To improve feature extraction, the model incorporates a distinct CNN‐based DAMFN track alongside the Swin transformer track. The DAMFN track specializes in low‐level feature extraction, whereas the Swin transformer track focuses on high‐level feature extraction. After merging the data from the two tracks and running it through TA to highlight the important sections, global average pooling is utilized to reduce dimensions. Finally, the classification output is refined by applying a Cross‐Entropy loss‐optimized classification layer.

In the study by Tabbakh and Barpanda (2023), a new method for diagnosing damaged plant leaves and obtaining detailed information is provided. Plant diseases have a negative impact on the agriculture industry, causing crop and financial losses. Because traditional methods can be costly and time‐consuming, accurate and prompt diagnosis is critical for managing and controlling plant diseases. Deep learning‐based algorithms can successfully identify plant diseases if the collected data is of high quality. In this context, a hybrid model based on a vision transformer (TLMViT) and a Transfer Learning‐based model is proposed for the classification of plant diseases. The study by Ahmed et al. (2025) looks into how deep learning (DL) and transfer learning (TL) can be used with ResNet‐101 to enhance guava sickness diagnosis. The raw image data is sent directly into ResNet‐101, which can detect subtle patterns without requiring human feature extraction, resulting in an efficient and exact classification process.

Several common limitations have been found in examining the work related to crop disease diagnosis with deep learning. One persistent issue is the frequency of false positives and negatives, which point to the difficulties of precisely forecasting illness scenarios. Overfitting is one of the most persistent concerns because of using private or small datasets and inefficient training–testing splits. Moreover, visible concerns about selecting hyperparameters without experimenting with which value is optimized lack detailed justification, compromising model stability and robustness. The insufficient dataset size limits the models' generalizability, necessitating the acquisition of more extensive datasets. Many loopholes regarding the generalization to diverse crops, choosing a hyperparameters procedure, and usage of transfer learning most of the time instead of developing new models, which impact the model transparency and lack of comprehensive model comparisons, are recurrent themes. Moreover, there is concern about computational efficiency and scalability and a lack of detailed discussion. These limitations underscore the need for further improvement in data utilization and model generalization to advance the efficacy and transparency of deep learning models for diagnosing crop diseases in a practical environment.

## 3 Methodology

**FIGURE 1:**
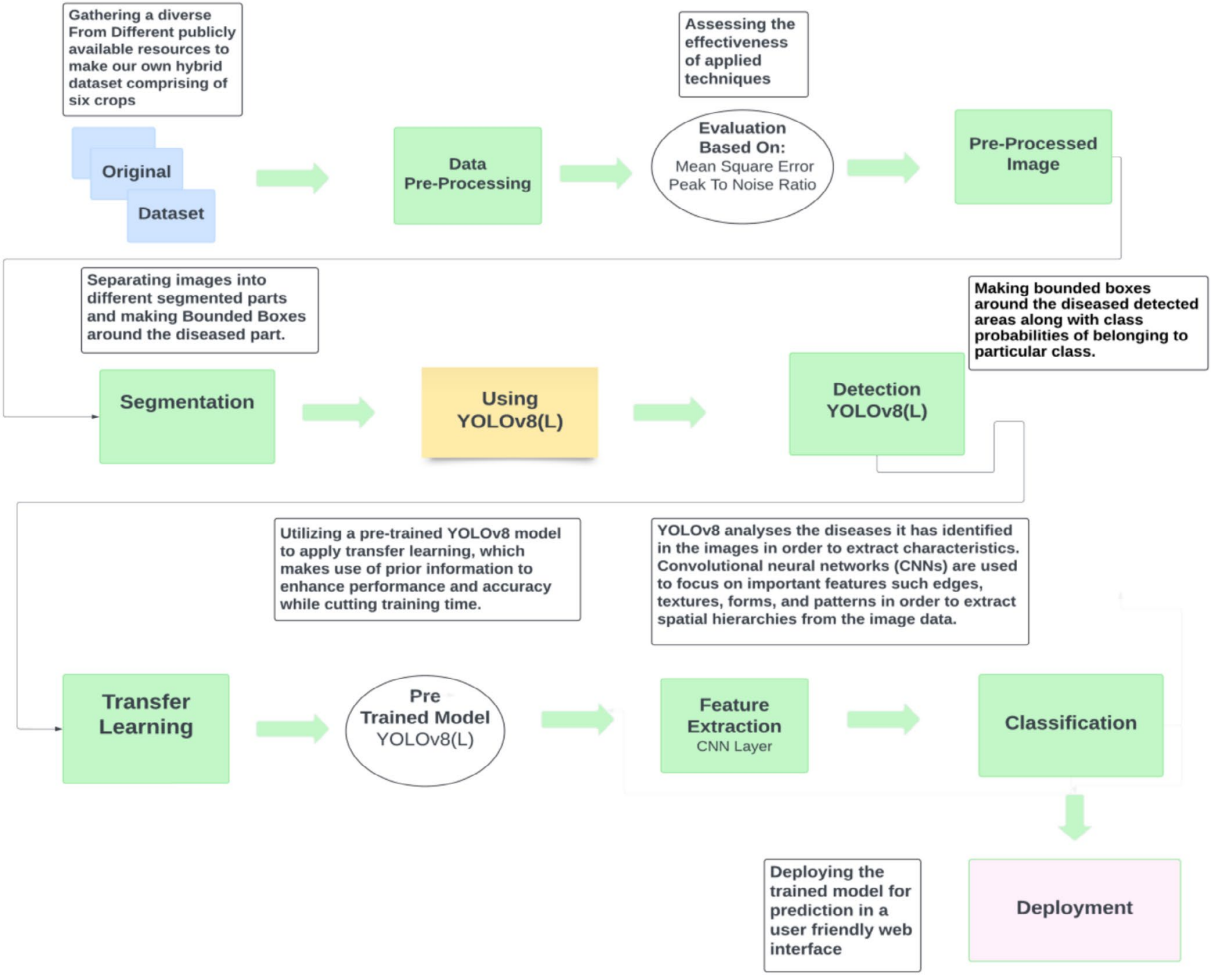
Methodology flow.

The methodology comprises a thorough approach to detect various diseases, primarily consisting of 32 diseases across six crops: cotton, cucumber, wheat, tomato, olive, and coffee. The start step involves gathering a hybrid and robust dataset from different reliable sources that are publicly available, subsequently carrying out broad pre‐processing on the collected data to ensure the data quality and consistency. Following pre‐processing, the crop images accurately marked the areas of interest and performed detailed annotation on the labeled data for further segmentation, detection, and classification purposes. Subsequently, contours are applied to fine‐tune the regions that have been segmented further, and then the segmented areas are delineated with precision. Utilizing the power of YOLOv8, a cutting‐edge deep learning model, to perform both disease detection and classification. Lastly, the model was deployed into a web‐based application, which made it more generalized and accessible for practical usage. The methodology flow is shown in Figure 1.

### 3.1 Dataset

**FIGURE 2:**
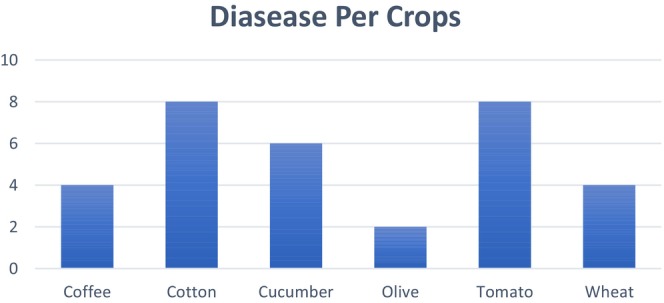
Classification schema for the six crops and their classes.

**FIGURE 3:**
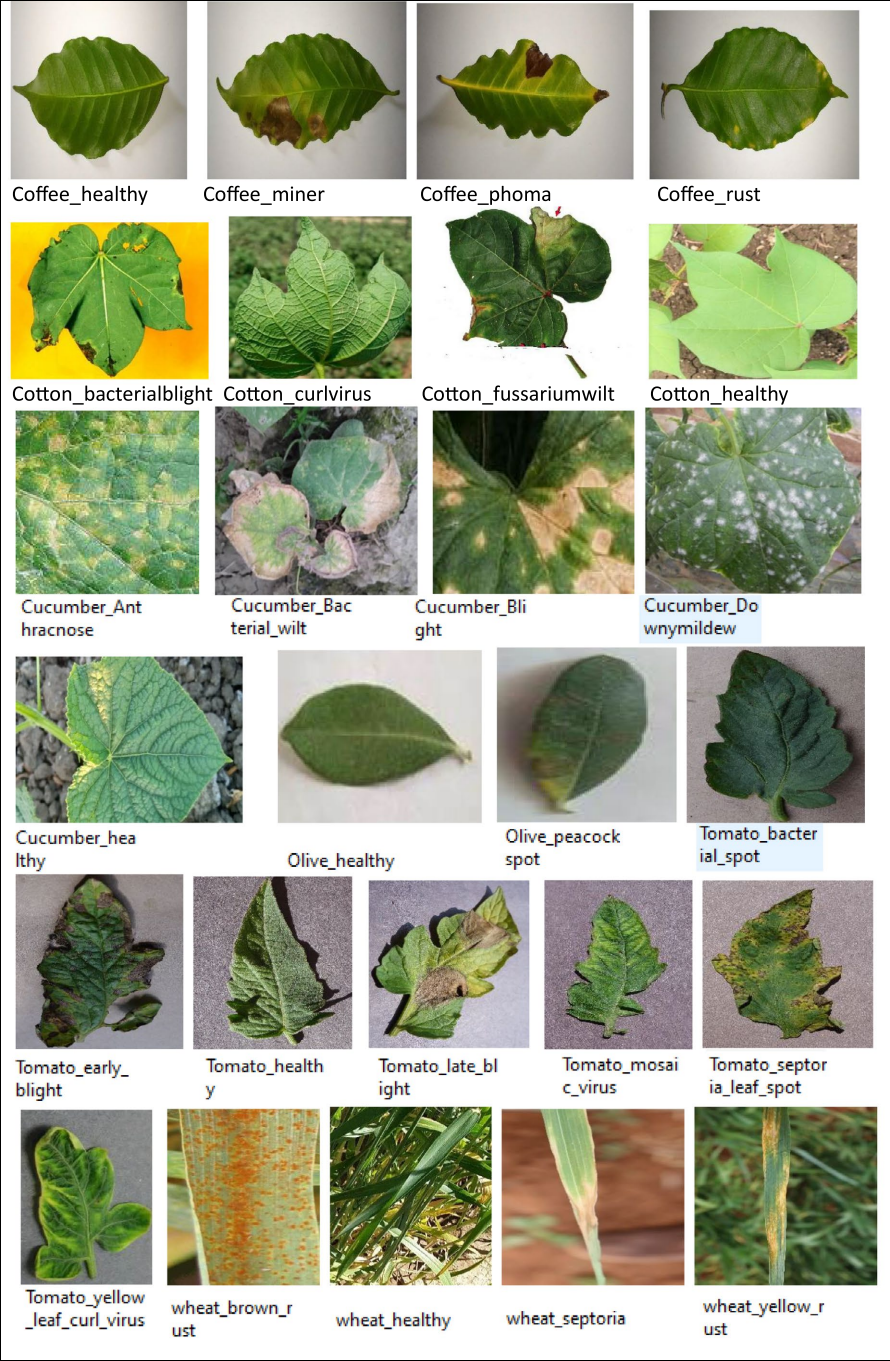
Sample images from the dataset for 32 types of crop diseases.

Our research utilizes a comprehensive, publicly available dataset designed for crop disease diagnosis. This hybrid dataset was collected from numerous reliable, publicly available sources, primarily consisting of six significant crops: cotton, cucumber, wheat, tomato, olive, and coffee. Subsequently, each crop is subdivided into diseases, consisting of 32 classes across six crops. The classes' distribution is as follows: coffee (four classes), cotton (eight classes), cucumber (six classes), olive (two classes), tomato (eight classes), and wheat (four classes). Initially, the dataset is imbalanced. To address this imbalance, data augmentation techniques were employed during pre‐processing, resulting in a balanced dataset with each class comprised of 500 images, which guarantees a more reliable and comprehensive approach for classifying crop diseases. The dataset was taken from PlantVillage, AI‐Hub, Embrapa, Wheat Rust Classification Dataset, and Changping. Figure 2 shows the details of the classes corresponding to each, while Figure 3 shows the sample images of the dataset.

### 3.2 Pre‐Processing

The original images collected from the dataset might contain noise, and it is imperative to pre‐process them before including them in the study module (Islam et al. 2023). Approximately nine pre‐processing techniques were applied to optimize image data for subsequent analysis. For evaluation, metrics like mean squared error and peak signal‐to‐noise ratio (PSNR) were used and found that the median filter was the most effective.

- *Gamma correction*: It is a nonlinear method typically used for modifying image contrast and brightness by efficiently increasing or decreasing overall brightness by applying a power‐law function to the pixel values, improving the image's visibility and detail perception. This method works very well for correcting luminance discrepancies and optimizing image quality for analysis.
- *Logarithmic transformation*: The logarithmic transformation technique involves taking the logarithm of each pixel's intensity value by lowering the intensity of the brighter parts while highlighting the details of the dark region simultaneously; this transformation effectively compresses the dynamic range of pixel values. Enhancing low‐contrast photos with a logarithmic transformation helps make subtle details more visible and makes image analysis chores easier in the future.
- *Local contrast enhancement*: This technique improves the image quality through the augmentation of local contrast enhancement. Local contrast enhancement techniques sharpen image features and improve texture visibility by applying filters or algorithms that magnify the differences between surrounding pixel values. Using a few methods, such as local and global illumination, is highly likely to effectively overcome the problem associated with misrepresented shadows or fuzzy images.
- *Wavelet transform*: Wavelet transform is typically a mathematical technique to separate or divide images into different frequency signals. Analyzing these frequencies individually effectively depicts a picture's minute details and broad structures. This approach is mainly used for denoising, compression, and feature extraction in image processing tasks to analyze various solutions.
- *Histogram equalization*: This technique changes the image's contrast by rearranging the intensity values over the histogram, extending the intensity range to screen the dynamic range of pixel values, and improving overall contrast and detail visibility. This technique benefits images with tilt or low contrast, resulting in more enhanced and visually pleasing images.
- *Sigmoid correction*: This is also a nonlinear technique typically used to extend or increase the pixel values in an image to efficiently increase the image's contrast while maintaining the overall tonal balance by mapping the input pixel values to a sigmoidal curve. This technique is primarily applied in remote sensing and medical imaging to enhance image quality.
- *Linear contrast adjustment*: This technique is used to extend the intensity range of an image linearly, using a linear transformation function to map the input intensity values to a new range of values, enhancing the image's overall contrast while preserving its original features (color). This method is primarily used in picture editing software to enhance the visual quality of images.
- *Unsharp mask filtering*: This technique, also known as the sharpening technique, is used to improve the edges and minor details in the images. It involves subtracting the vague version of the image from the original image, resulting in a more enhanced image along with edges. It improves the visual clarity of the image by efficiently highlighting the local intensity changes in an image, increasing its apparent sharpness.
- *Median filtering*: The research on median‐based filters has yielded remarkable results and highlighted some new, promising research avenues. Even though median filters are widely used in image processing, they tend to blur sharp edges, destroy lines and other fine image details, fail to effectively remove heavy‐tailed noise, and perform poorly in the presence of signal‐dependent noise. Because of their simplicity and edge preservation.

After applying the above‐discussed pre‐processing techniques, the median filter gives the best results compared to the other eight techniques based on qualitative and quantitative analysis of results. The median filter removes the noise in the images while preserving the edges of the images. Unlike other filters, it selects the middle value by examining a set of pixels surrounding each point rather than averaging the pixel values. This nonlinear filtering method works particularly well at minimizing impulse noise when there is little to no blurring of the image features. The median filter works well for various image‐enhancing tasks because it resists outliers and preserves image sharpness.

Table 1 shows the qualitative analysis of the applied pre‐processing technique, and Table 2 shows the applied technique's quantitative results, which were subsequently evaluated based on peak‐to‐noise ratio (PSNR) and mean squared error (MSE). In Table 3, a comparison of the PSNR and MSE values of applied preprocessing techniques is shown to highlight the importance of picking the median filtering.

**TABLE 1:** Qualitative analysis.

Crops	Label	Before	After
Coffee	Coffee_rust	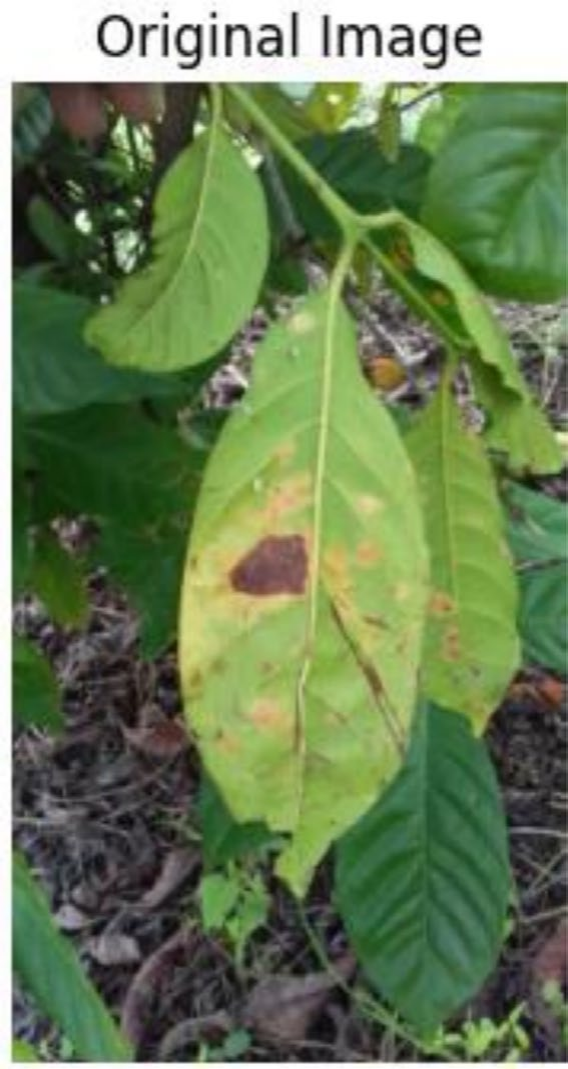	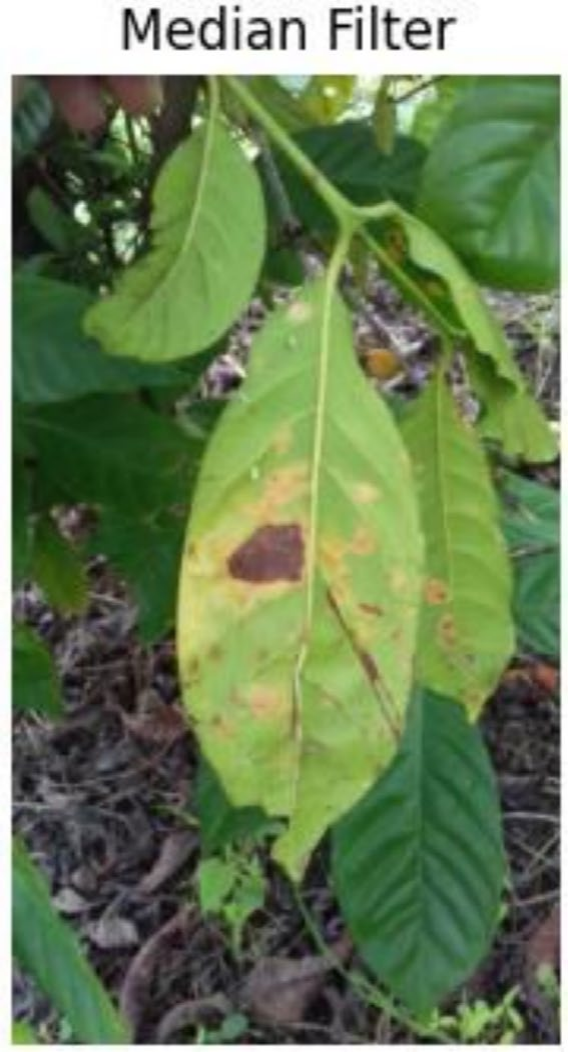
Wheat	Wheat_brown_rust	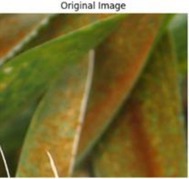	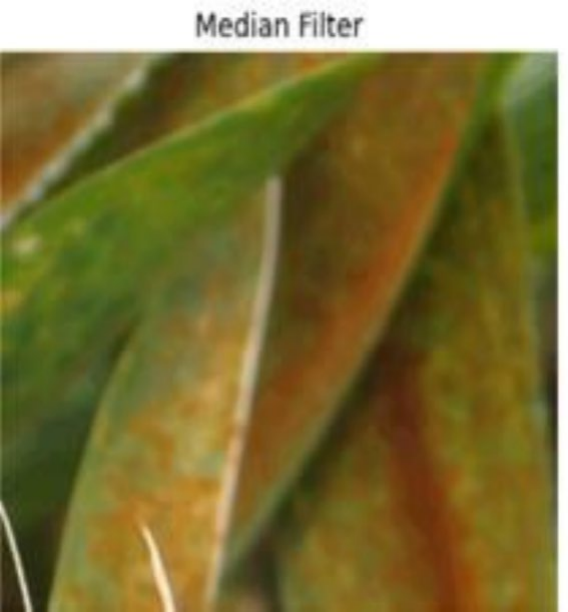
Cotton	Cotton_bacterial_blight	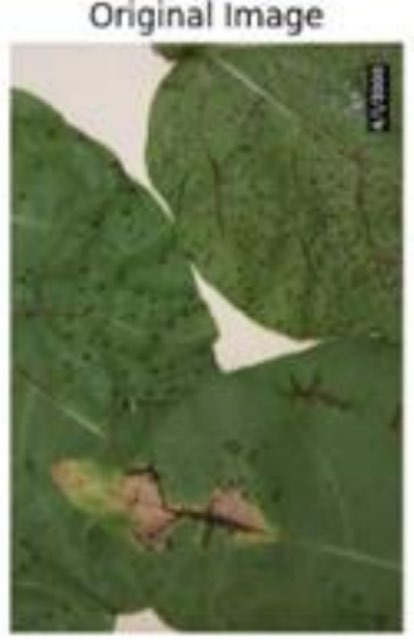	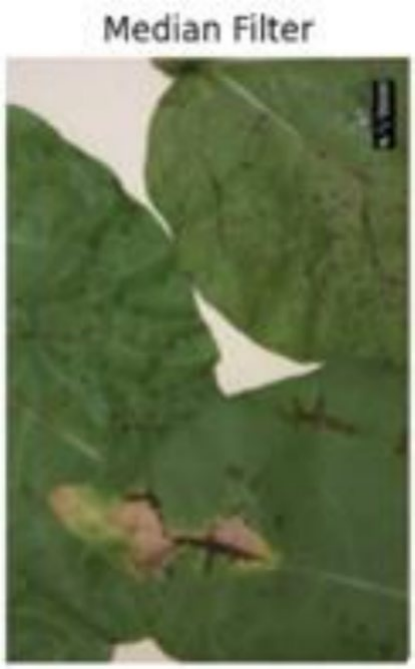
Cucumber	Cucumber_anthracnose	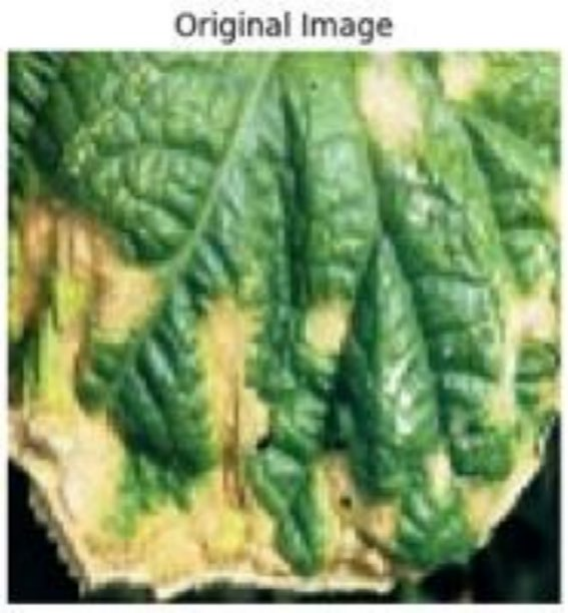	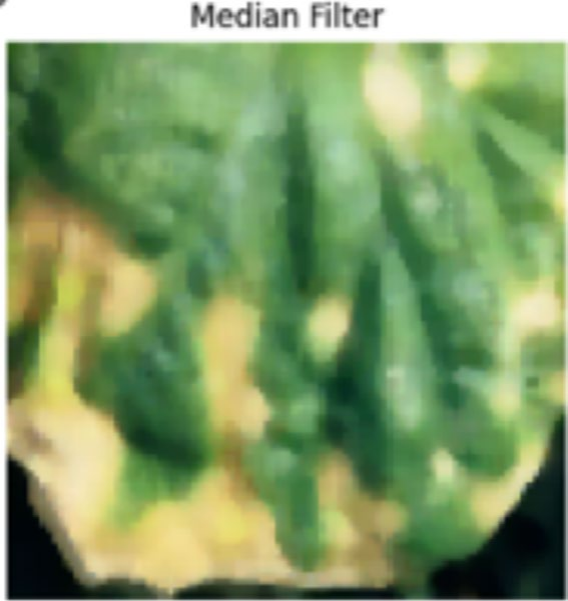
Olive	Olive_peacock_spot	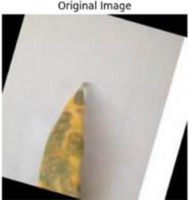	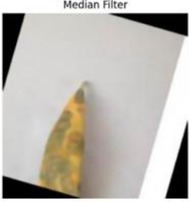
Tomato	Tomato_bacterial_spot	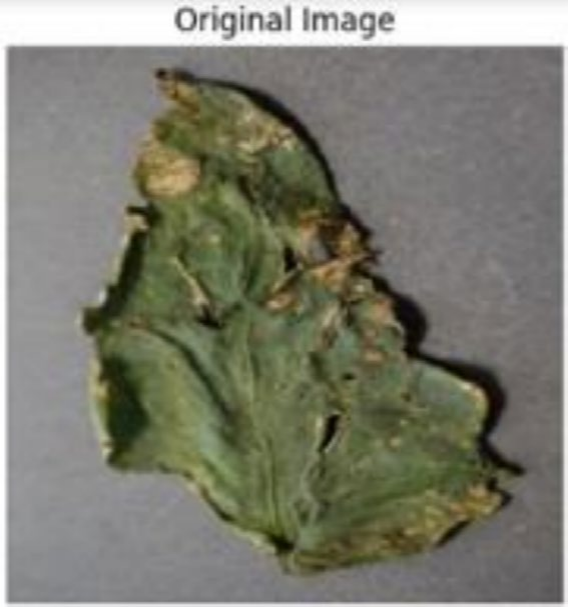	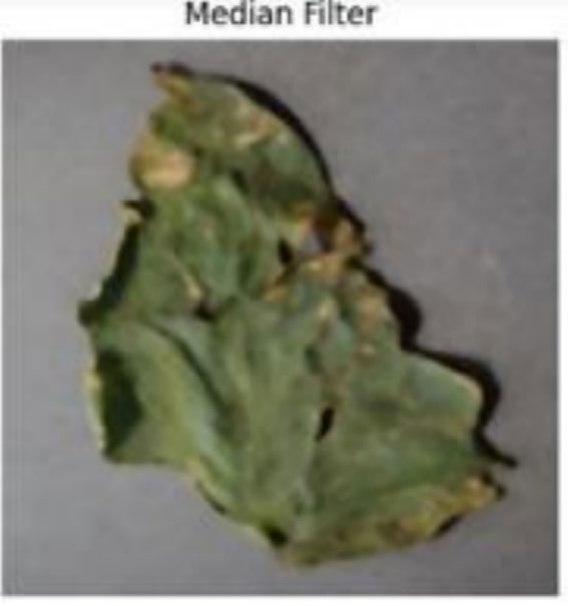

**TABLE 2:** Quantitative analysis of median filtering.

Crops	Label	PSNR value	MSE value
Coffee	Coffee_rust	38.31125	19.59300
	Coffee_healthy	37.21112	20.87901
	Coffee_miner	36.23145	19.33121
	Coffee_phoma	38.66547	19.71642
Wheat	Wheat_brown_rust	35.38936	18.79940
	Wheat_healthy	37.98112	19.11735
	Wheat_yellow_rust	39.01047	18.11723
	Wheat_septoria	38.66312	17.63442
Cotton	Cotton_curlvirus	37.28110	19.98316
	Cotton_fussariumwilt	34.34989	20.10984
	Cotton_healthy	39.32163	17.00013
	Cotton_bacterial_blight	35.28739	19.24603
Cucumber	Cucumber_Anthracnose	40.38541	16.74990
	Cucumber_Bacterial_wilt	41.77004	16.01033
	Cucumber_Blight	40.21255	17.13224
	Cucumber_Downymildew	39.10201	18.04129
	Cucumber_healthy	43.60998	21.83192
Olive	Olive_peacock_spot	42.61756	31.55896
	Olive_healthy	40.70119	28.12231
	Olive_bacterial_spot	38.98116	24.13768
Tomato	Tomato_late_blight	34.48664	27.14280
	Tomato_yellow_leaf_curl_virus	39.40712	18.01073
	Tomato_early_blight	38.56314	17.46431
	Tomato_healthy	37.18015	19.18417
	Tomato_mosaic_virus	34.64887	20.51086
	Tomato_septoria_leaf_spot	41.87091	16.11434

**TABLE 3:** Comparative analysis of preprocessing techniques.

Preprocessing techniques	PSNR	MSE
Gamma correction	28.30124	118.90301
Logarithmic transformation	27.63245	97.49076
Local contrast enhancement	21.98723	213.34801
Wavelet transform	23.10988	190.12319
Histogram equalization	29.64118	57.35565
Sigmoid correction	30.15672	54.13984
Linear contrast adjustment	31.99281	51.63671
Unsharp mask filtering	29.76193	61.44581
Median filtering	38.83512	22.87129

### 3.3 Segmentation

The image segmentation technique divides the image into several segments to separate areas of interest where a particular disease is present. Particularly in crop disease segmentation, the research aims to locate and define the pale areas within the crop leaves with a more focused target analysis and diagnosis. Subsequently, this enables easier examination of specific features and the severity of the diseases impacting different crops. YOLOv8, although primarily known for its object detection capabilities, has also proven to be significantly effective in crop disease detection. It leverages Convolutional Neural Networks (CNNs) to analyze images and predict pixel‐wise segmentation masks. YOLOv8 possesses the quality of passing entire images in a single forward pass, which makes it computationally efficient and exceptional for real‐time applications. Segmentation masks produced by the model highlight image parts containing the disease. Subsequently, this integrated approach results in the efficiency and accuracy of the model in identifying and demarcating diseased areas.

The dataset was initially arranged to meet the input sequence criteria of YOLOv8, aligning the images and accompanying annotations in a way compatible with the YOLOv8 format before segmentation was performed. After experimenting with several variants of YOLOv8, which include nano, small, large, and extra‐large, the YOLOv8 large variant was used; it shows prominent results, as the dataset was large and complex enough to use. A pre‐trained YOLOv8 large segmentation model was loaded, featuring weights trained on various image sets. This step is significant as it gives the model a good foundation to expand upon previously acquired features and patterns. The training process then started to ensure the efficiency, generalizability, and precision of the epochs set to run 50 to fine‐tune the model on the particular dataset. During the initial training phase, we did not freeze the backbone weights. The entire YOLOv8 model, including the backbone, neck, and detection head layers, was fine‐tuned on our custom dataset. We adopted this approach as recent studies suggest that full fine‐tuning with a lower learning rate can yield substantial performance gains for fine‐grained or specialized tasks without significant performance degradation on general knowledge. The model learns to segment and identify accurately diseased parts within the crop's images during training. As training ends, the testing process starts to evaluate the model's performance. Testing scores corresponding to each image were obtained, which ensures the exhaustive assessment of the model's accuracy and robustness in segmenting diseased regions across the entire dataset.

After segmentation is applied, contours are drawn around the boundary regions using the Convex Hull approach. To represent the general outer boundary of a segmented lesion, the Convex Hull method was applied. A convex hull is defined as the smallest convex shape that completely encloses all the points on the boundary of the lesion. It can be visualized as a rubber band stretched around the lesion's outermost points, forming a smooth contour that eliminates small irregularities caused by noise or segmentation artifacts. Thus, studying the convex hull helps provide quantitative descriptors that can aid in classification.

### 3.4 Detection

Detection involves verifying the presence and classifying the segmented diseased areas within the crop. This process involves training the entire annotated dataset and model to identify or classify diseased regions accurately. After training, the model analyzes the image of a particular crop and makes bounded boxes around the diseased areas, along with class probabilities of belonging to a specific class. The YOLOv8 pre‐trained model is loaded and fine‐tuned, and the large variant of YOLOv8 has been used as it is well‐suited to the dataset after being experimented with the other variants. Subsequently, the training starts and epochs are set to 50; the model starts learning to identify the diseased regions during training. After training, the testing process begins; it outputs or makes the bounded boxes across the diseased area and is classified into different classes along with the class probabilities (testing‐score), likelihood of each specific region belonging to a particular class, providing insights into its accuracy and effectiveness in crop disease detection.

**FIGURE 4:**
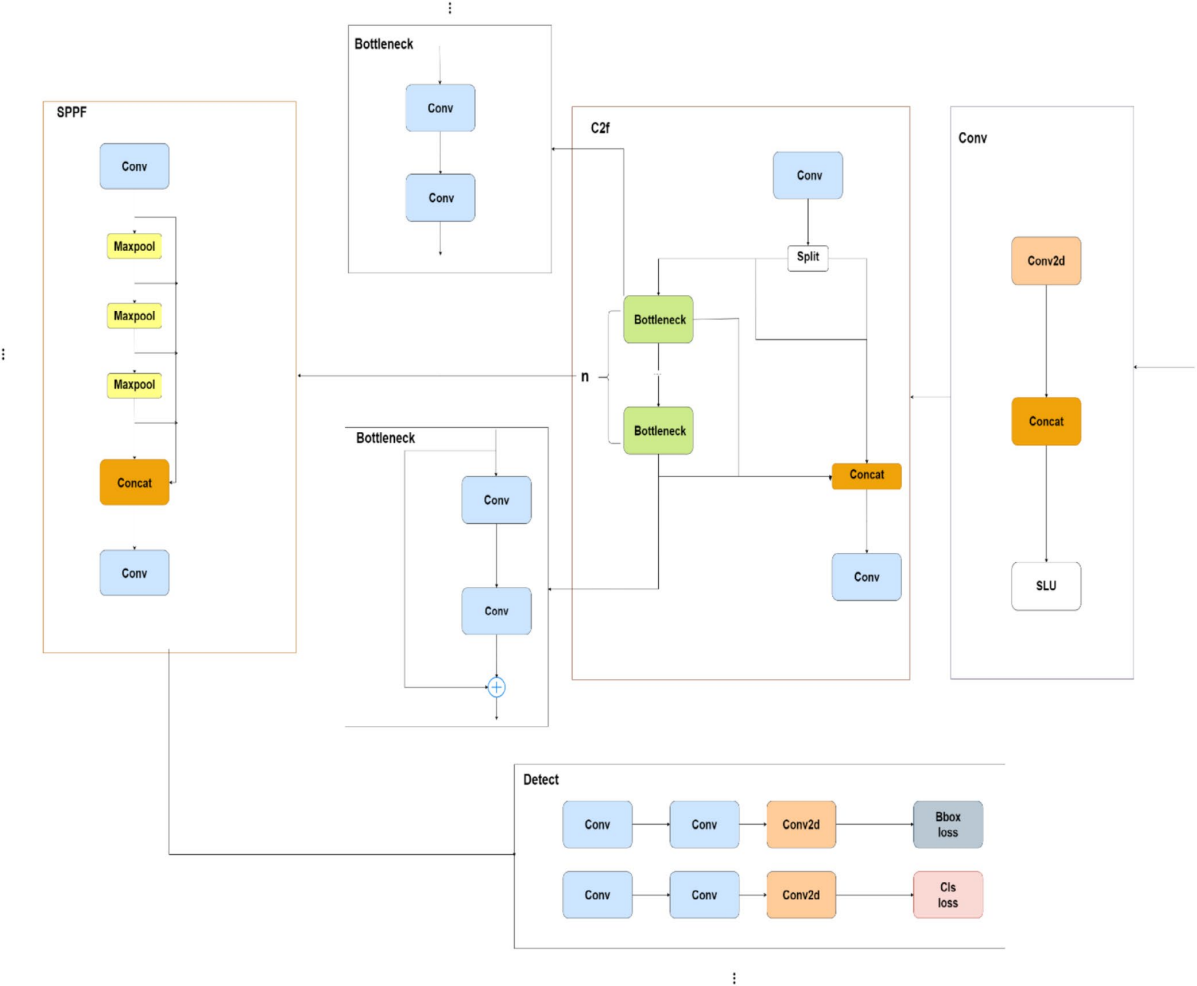
YOLOv8 architecture.

Figure 4 illustrates the work of YOLOv8 adopted for the detection of diseases in crops. The SPPF module first performs max‐pooling and convolution on the input image to extract significant spatial features at various scales. After that, these characteristics are routed through several bottleneck layers that preserve gradient flow through residual connections, aiding in learning complex representations. The C2f module divides the data into several channels for further processing, enabling the network to gather several combined feature sets. Subsequently, the Detect module enhances these characteristics and completes the remaining prediction tasks, such as classification and bounding box regression. The YOLOv8 model's sophisticated architecture enables it to effectively execute segmentation, detection, and classification tasks, which makes it an optimal choice for particular detection, distinguishing different plant diseases from the given dataset.

### 3.5 Classification

Classification involves allocating labels according to the dataset to each detected and segmented region, and the features extracted by the model are used to identify the type or class of a particular disease present in the cropped image. Although YOLOv8's primary purpose is object detection, it also shows prominent results in classifying crop diseases by examining the features within bounding boxes detected earlier. After experimenting with all variants of YOLOv8, a large variant of YOLOv8 is selected because of its superior performance and suitability to the dataset; YOLOv8 processes the image in a single forward pass, which makes it more efficient while extracting the features and patterns using the convolutional layers and classifying the detected objects.

For classification purposes, the dataset is arranged according to the input sequence of the YOLOv8 model, and then the pre‐trained model YOLOv8 variant large is loaded. The training starts by setting the epochs to 50 to optimize the model parameters, ensuring accurate disease prediction. The testing process begins by applying a trained model to new, unseen images or data for validation to evaluate model performance. This subsequently results in predicting the disease in each cropped image using the segmented and detected areas. Prediction plays a significant role in accurate diagnosis and efficient crop disease treatment.

## 4 Results

### 4.1 Segmentation

**FIGURE 5:**
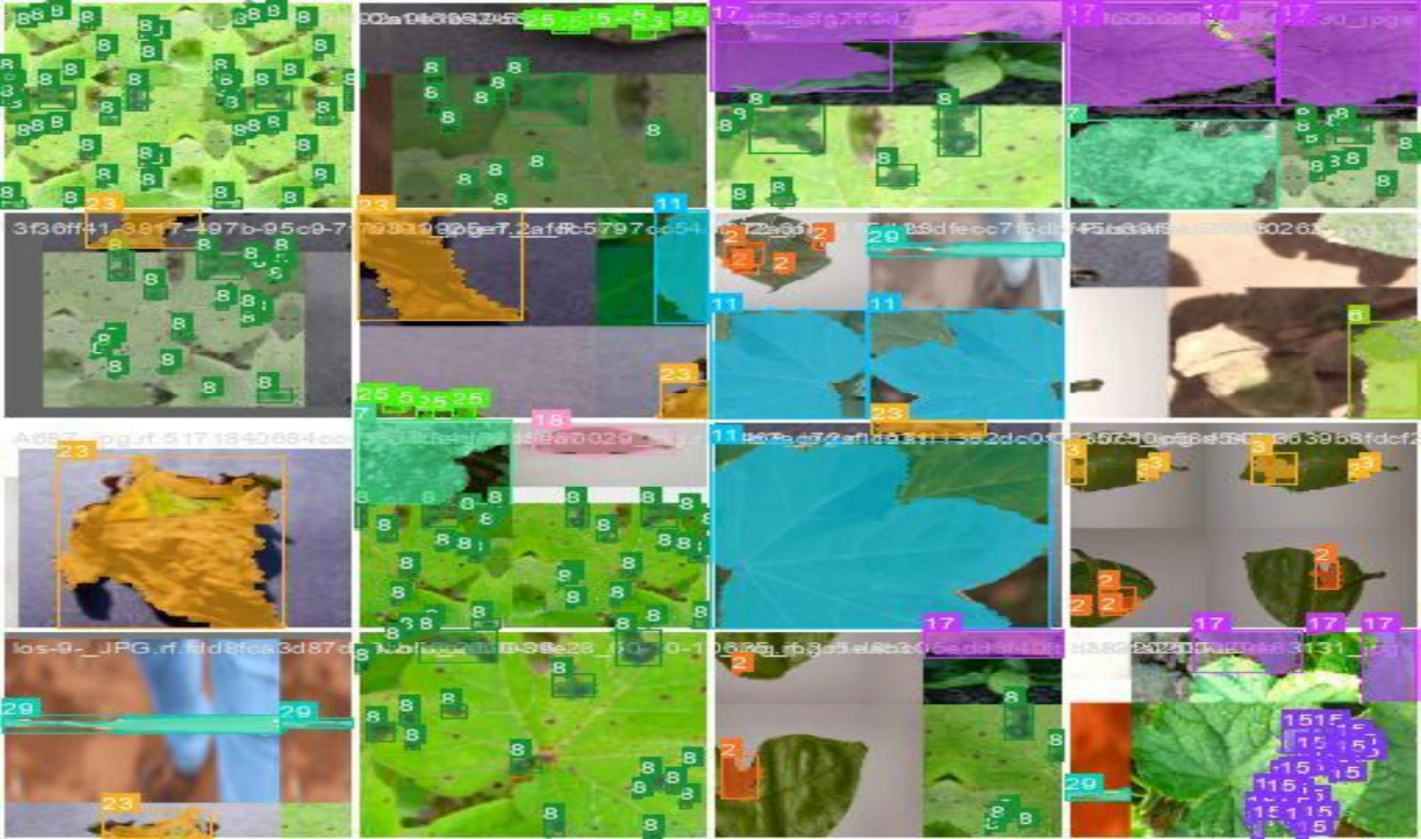
Training_batch 0.

Figure 5 shows the training process in segmentation, which is applied to the annotated images. The segmentation model recognized and labeled the crop leaves' various sick areas during training, and corresponding bounded boxes were created, colored boxes, and numbered to indicate the different illness classifications that the model can recognize. The green boxes illustrate the model's learning process, which shows the regions the model detected during training.

**FIGURE 6:**
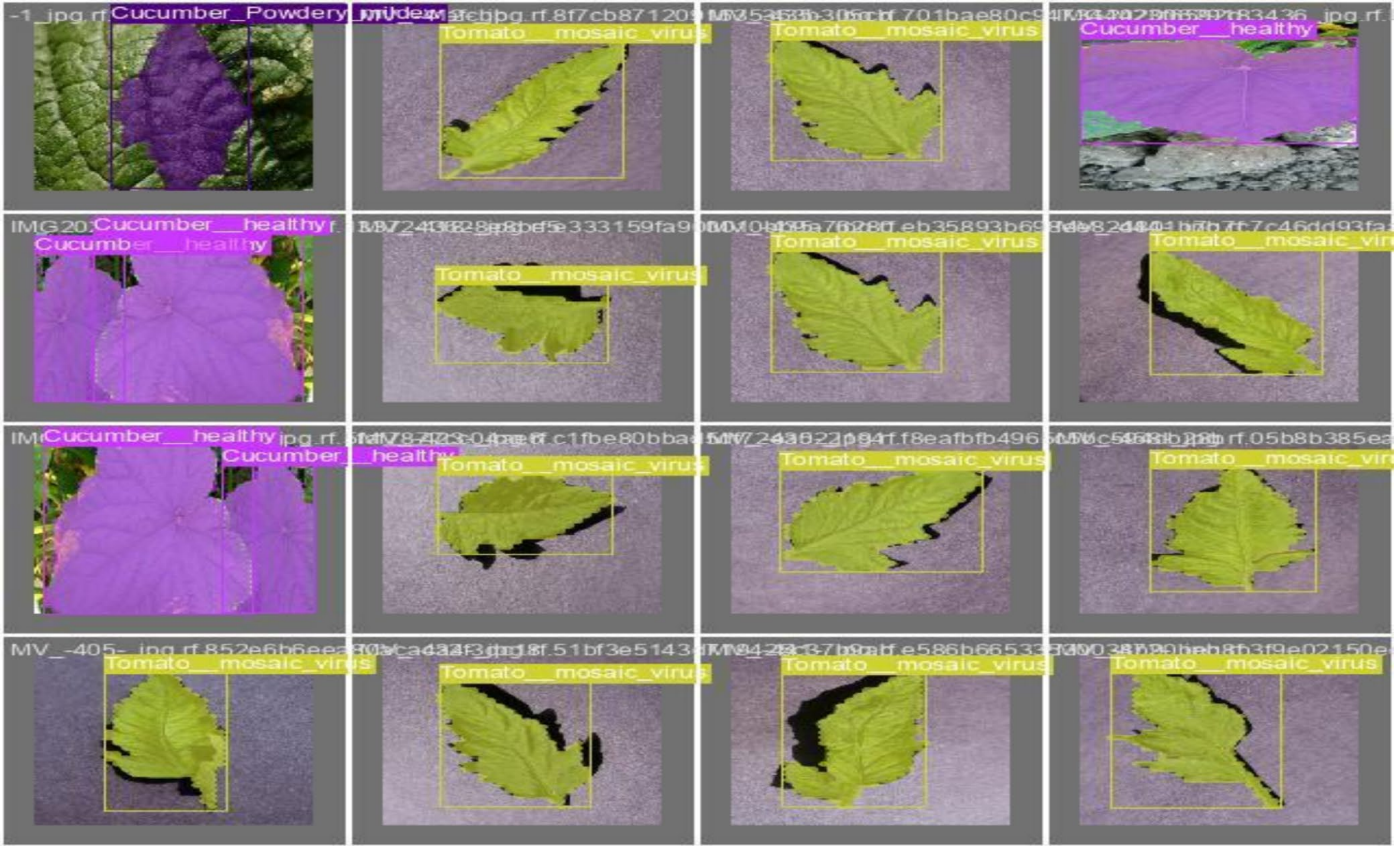
Validation of batch 0 labels.

Figure 6 shows the validation of the actual labels of batch 0 by evaluating the actual labels with the predicted ones. This step assesses the model's performance. The regions labeled in the validation dataset are highlighted by the overlays in purple, green, and other colors, allowing for the visual comparison to determine how well the model performed.

**FIGURE 7:**
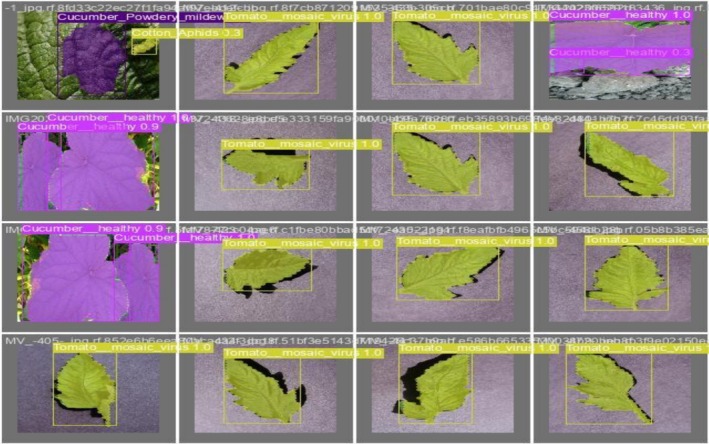
Validation of batch 0 prediction.

Figure 7 shows the validation of batch 0 prediction against the actual labels. In this figure, colored boxes and labels denoting the anticipated disease classes show the segmentations predicted by the model. An evaluation of the model's performance in practical situations is possible by contrasting these predictions with the validation labels. The model's capacity to generalize from the training data to fresh, unseen samples is shown by the correct diagnosis of illnesses like Cucumber Powdery Mildew and Tomato Mosaic Virus.

**FIGURE 8:**
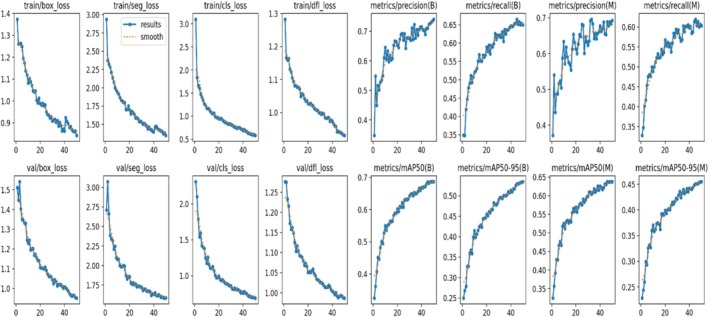
Various results of segmentation.

In Figure 8, a decrease in training and validation losses for the YOLOv8 model indicates significantly improved performance for segmentation tasks. The training loss measurements had persistent downward trends (train/box_loss, train/seg_loss, train/cls_loss, and train/dfl_loss). The train/seg_loss, train/cls_loss, train/dfl_loss, and train/box_loss all saw drops from 3.0 to 1.4, 3 to 0.6, and 1.3 to 0.5, 1.4 to 0.5, respectively. This pattern was reflected in the validation losses (val/box_loss, val/seg_loss, val/cls_loss, and val/dfl_loss), which decreased from 1.5 to 0.6, from 2.72 to 1.5, from 2.0 to 0.8, and from 1.2 to 0.9. There were also notable improvements in the measures for masks' precision and recall (metrics/precision [M] and metrics/recall [M]). Recall increased from 0.35 to 0.60 and precision from 0.35 to 0.70, respectively, indicating that the model could segment diseased regions more precisely. The average precision metrics for masks showed a consistent rising trend, both at the 50% intersection of the union threshold (metrics/mAP50 [M]) and at different thresholds (metrics/mAP50‐95 [M]). The metric shows that the model successfully segmented crop diseases when the mAP50 for masks grew from 0.35% to 0.70%, and the mAP50‐95 for masks increased from 0.25% to 0.45%.

**FIGURE 9:**
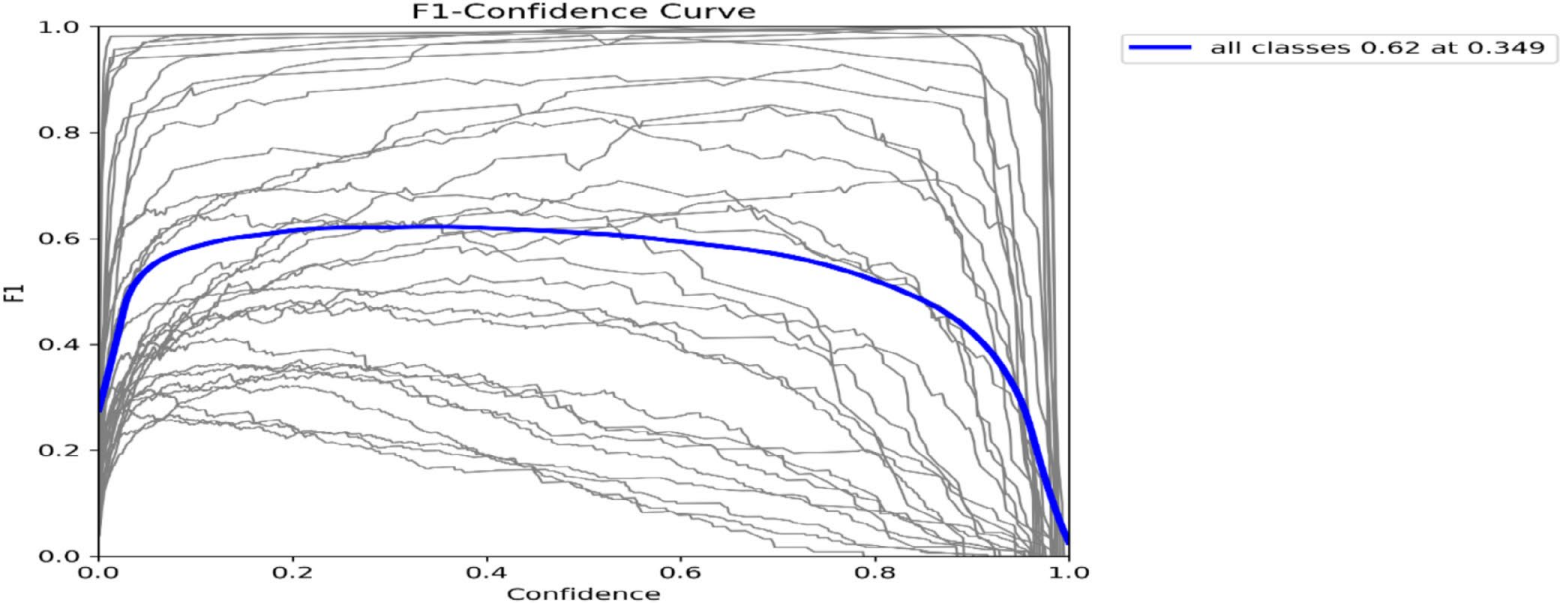
Mask F1 curve.

In Figure 9, the relationship between the F1 score and the confidence levels for each class is displayed by the F1‐confidence curve. At a confidence level of 0.349, the model obtains an F1 score of 0.62. The harmonic mean of recall and precision is reflected in the F1 score, which denotes a balanced performance. This curve contributes to finding the optimal threshold between precision and recall.

**FIGURE 10:**
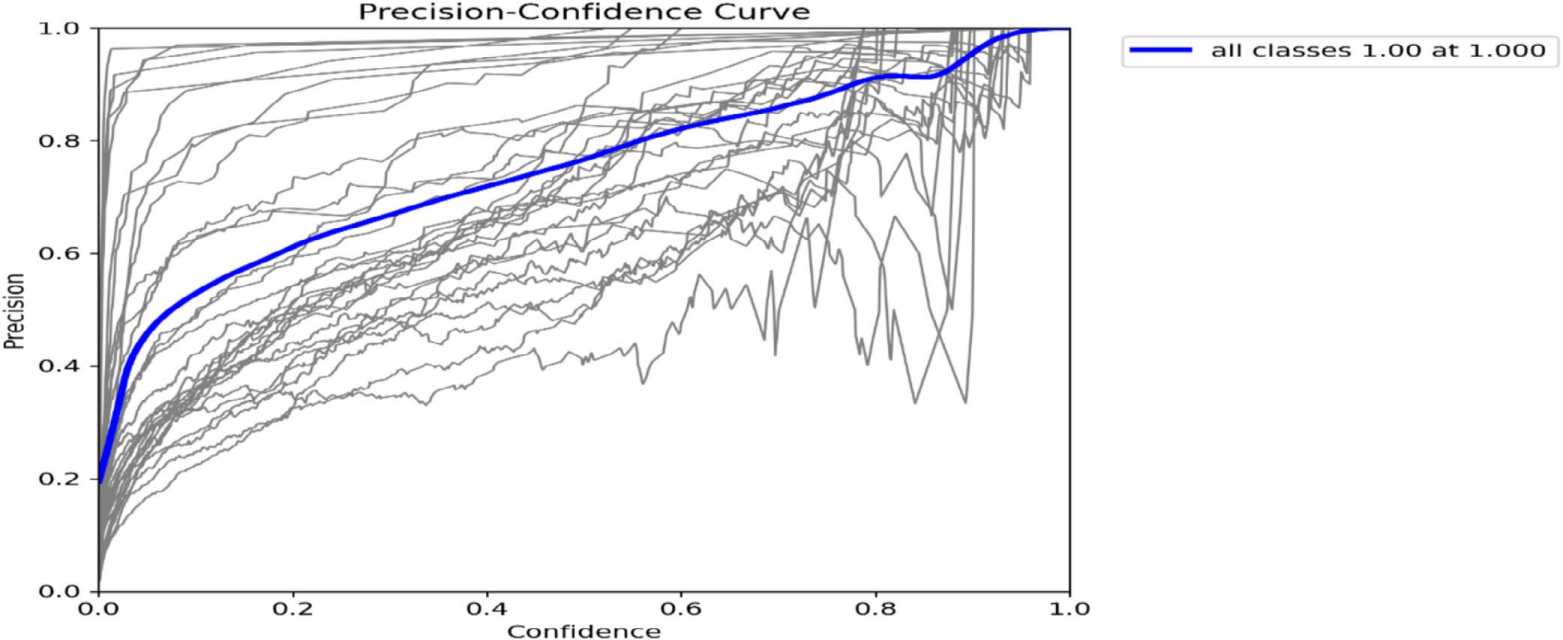
Mask precision curve.

Figure 10 shows the precision versus confidence levels on this curve. At a confidence level of 1.000, the model attains a precision of 1.00, signifying flawless perfection at this degree of assurance. Thus, every section predicted with this degree of confidence was accurate. However, precision alone does not account for false negatives, so it must be considered alongside recall.

**FIGURE 11:**
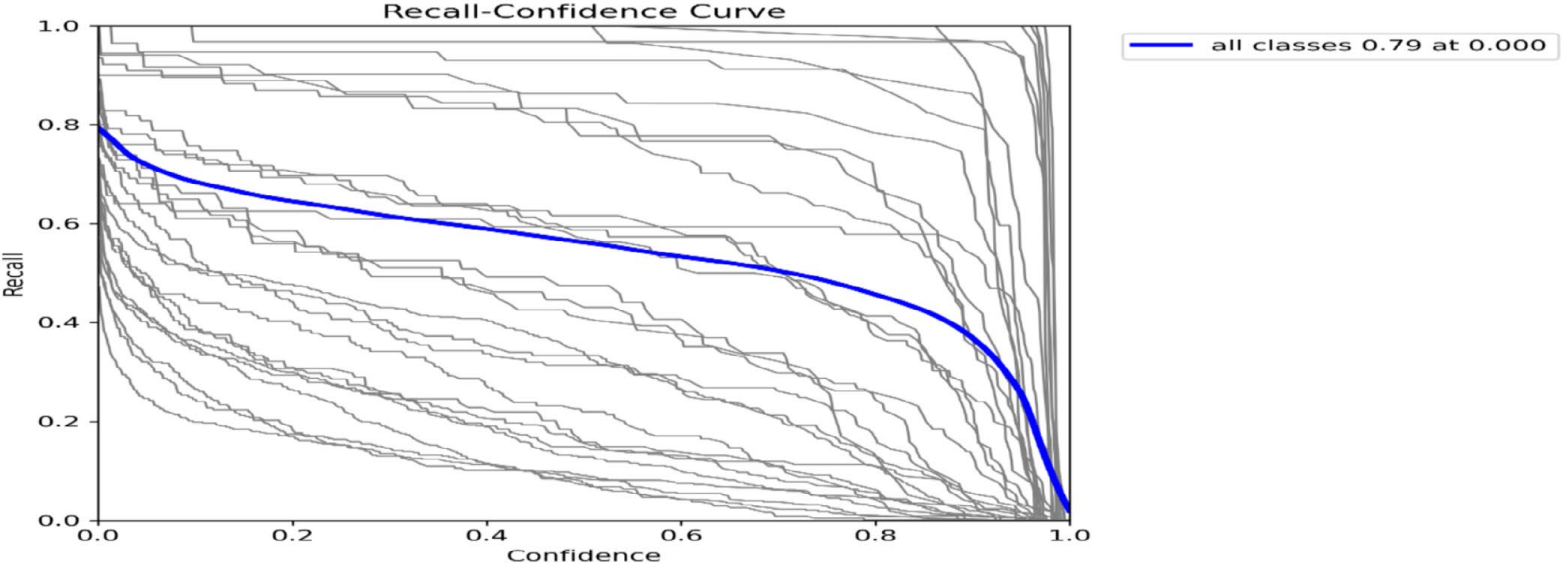
Mask recall curve.

Figure 11 recalls across the confidence illustrates that with a confidence level of 0.000, the model obtains a recall of 0.79, meaning that a significant percentage of real segments are correctly identified. A model with a high recall and low confidence level may be sensitive but may also contain false positives.

**FIGURE 12:**
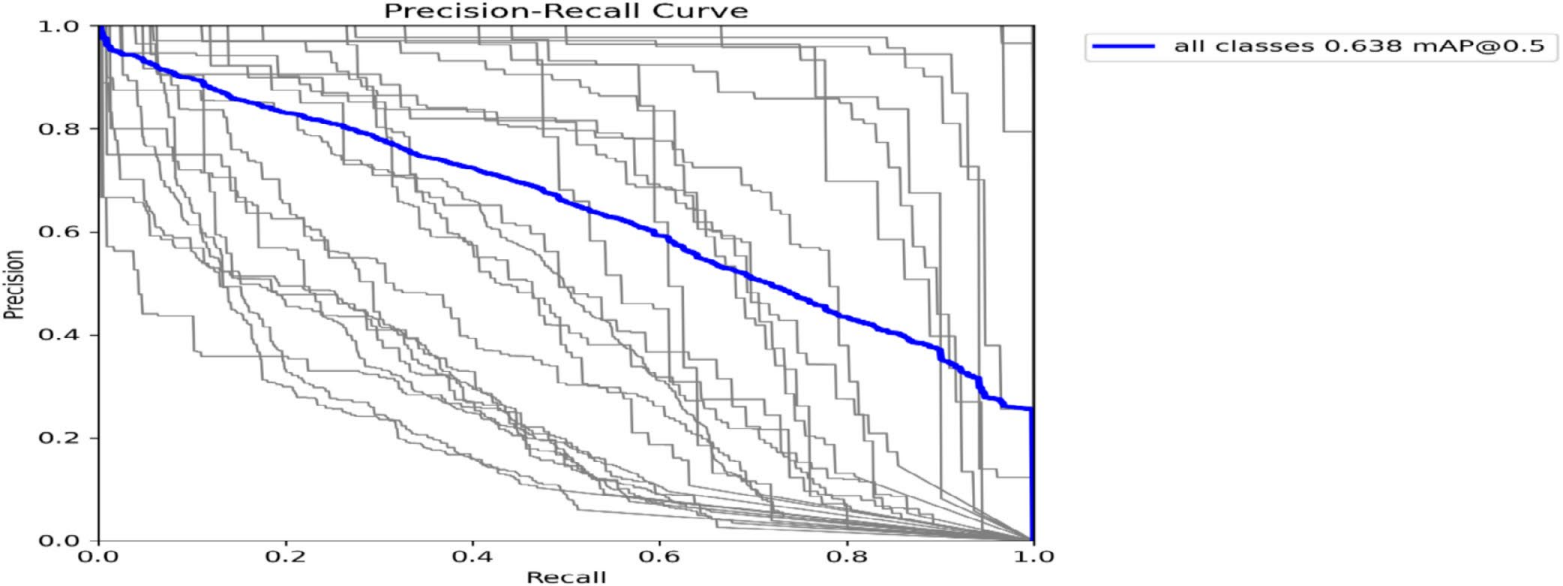
Mask precision‐recall curve.

Figure 12 shows a good balance between the two metrics as the model initially delivers high precision, approximately close to 1.0, while showing a trade between precision and recall. However, it gradually decreases later on, which is a typical tradeoff. Excellent performance is indicated by the model's mean average precision (mAP) of 0.638 at a threshold of 0.5, which it maintains while attempting to catch additional true positives. This curve, taken as a whole, shows that the model is balanced in its prediction powers and operates effectively.

**FIGURE 13:**
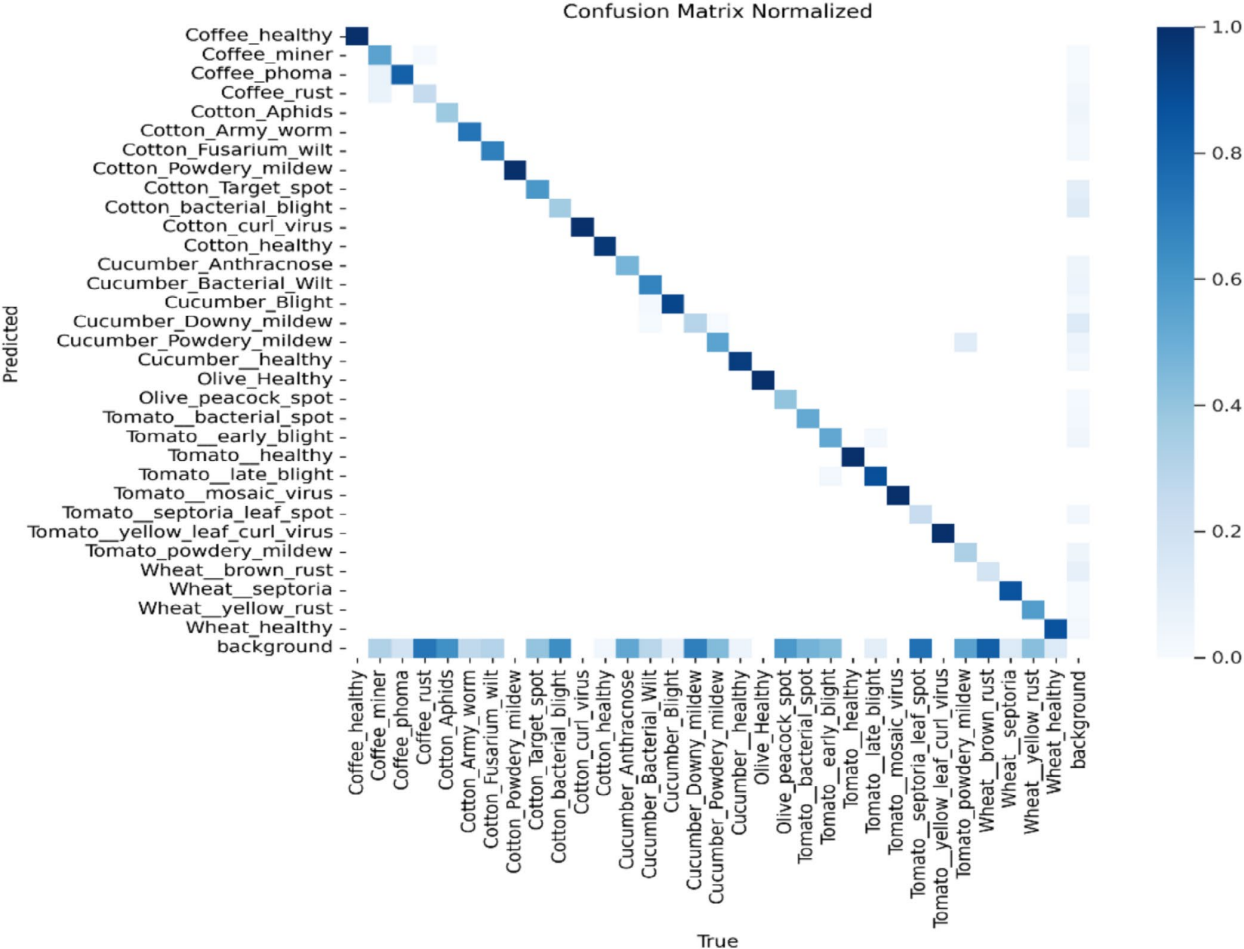
Confusion matrix.

Figure 13 shows the performance of the segmentation model under various plant illnesses. The normalized frequency of predictions is demonstrated by each cell, and the diagonal elements display the percentage of accurately predicted cases for each class. As shown in the figure, the model performs better for the classes that are darker in color. At the same time, there is also inevitable misclassification for the classes, but the blue color shows that the model performs well in all the classes. The lighter hues off‐diagonal in some categories, such as “Cotton_Aphids” and “Tomato_bacterial_spot,” indicate some confusion between these classes.

### 4.2 Detection

**FIGURE 14:**
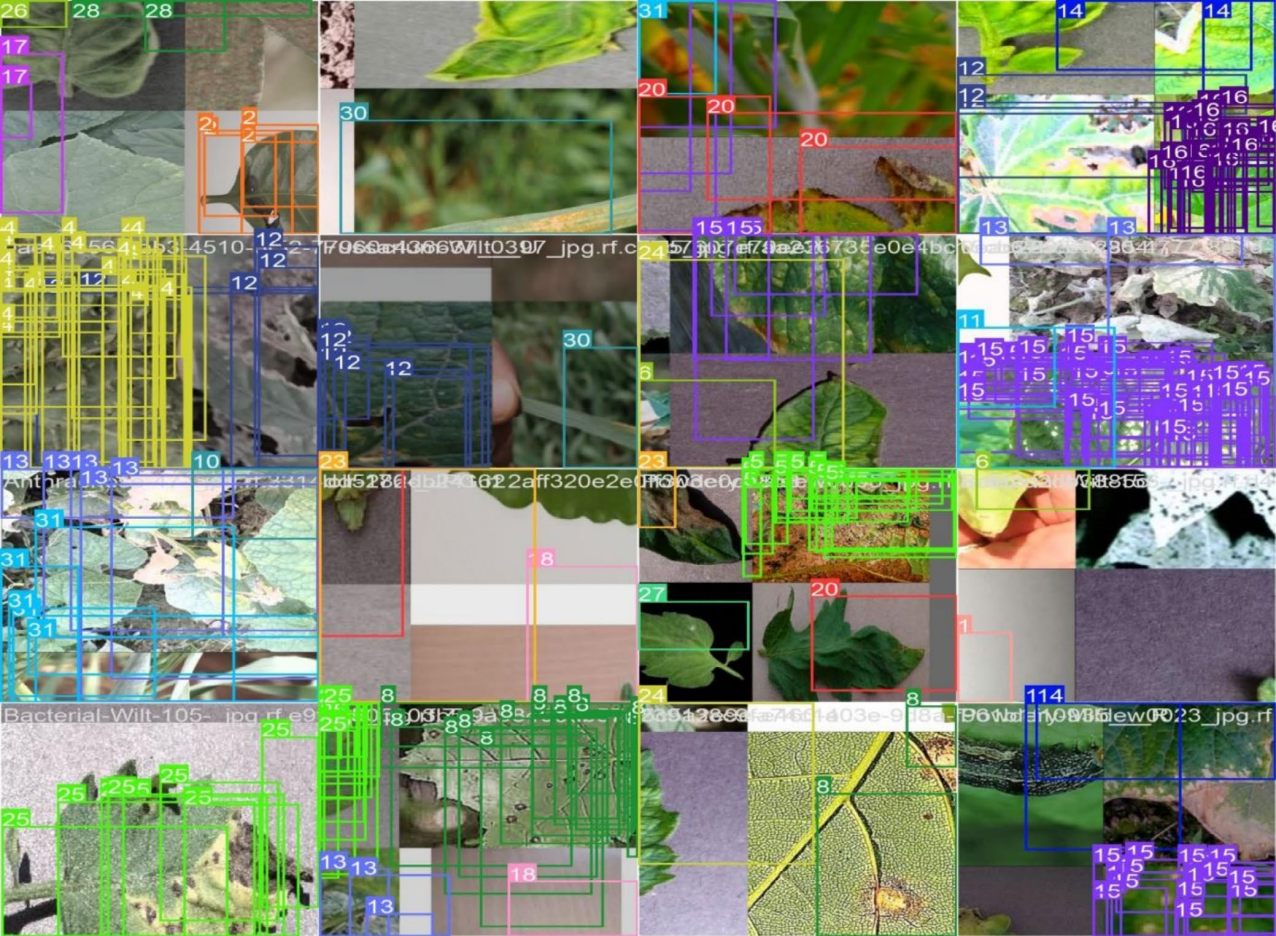
Training batch 0.

Figure 14 shows the training of batch 0 for detection using YOLOv8; the figure shows several bounding boxes in different colors, each representing a distinct class of detected objects. These boxes show the model's predictions made during the first training phase. They are labeled with class identities and confidence scores. Also, the overlapping bounded boxes show that the model is trying to learn to differentiate between multiple instances. This figure shows the YOLOv8 model's performance at batch 0, emphasizing its early object localization capabilities.

**FIGURE 15:**
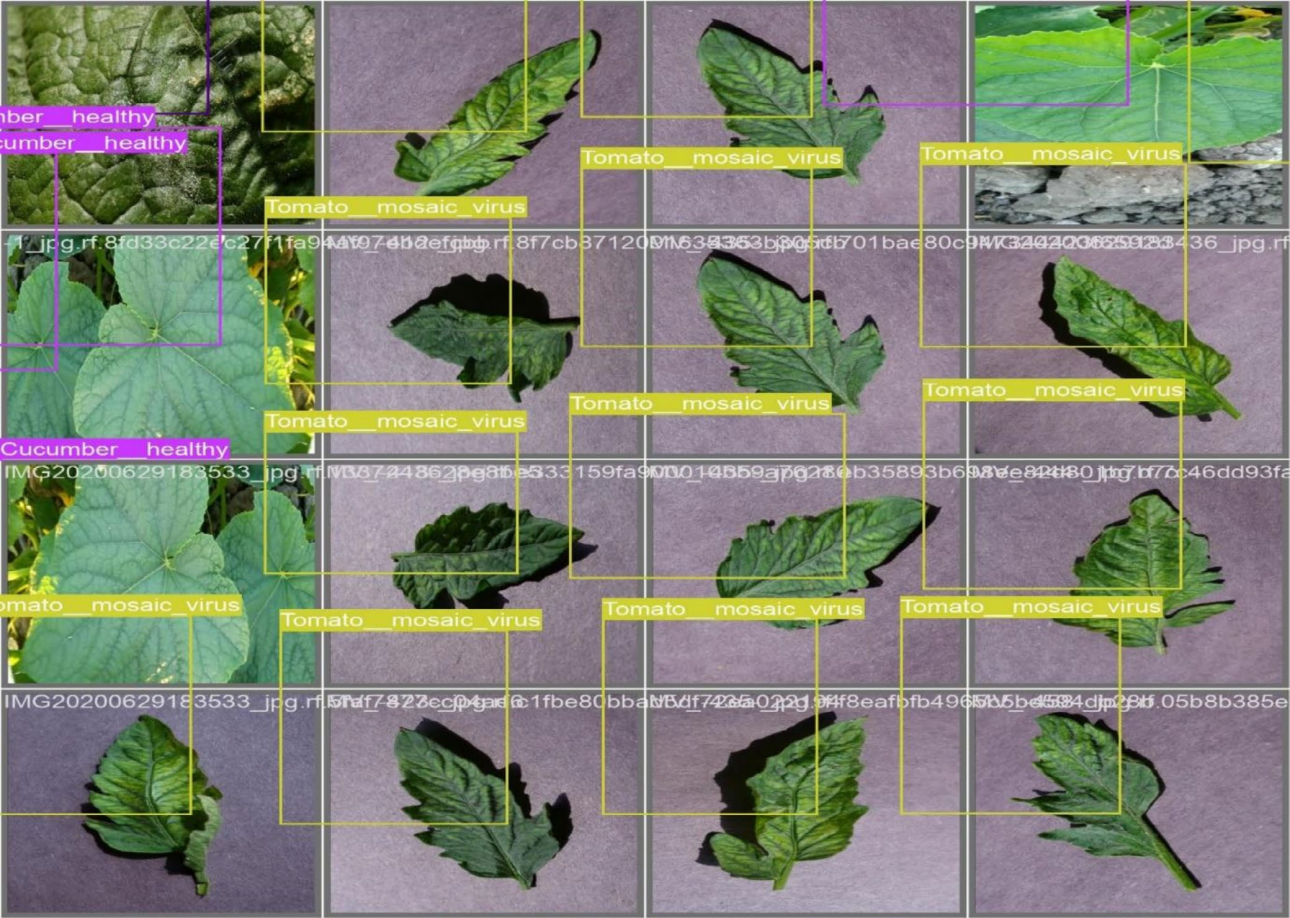
Validating batch 0 labels.

**FIGURE 16:**
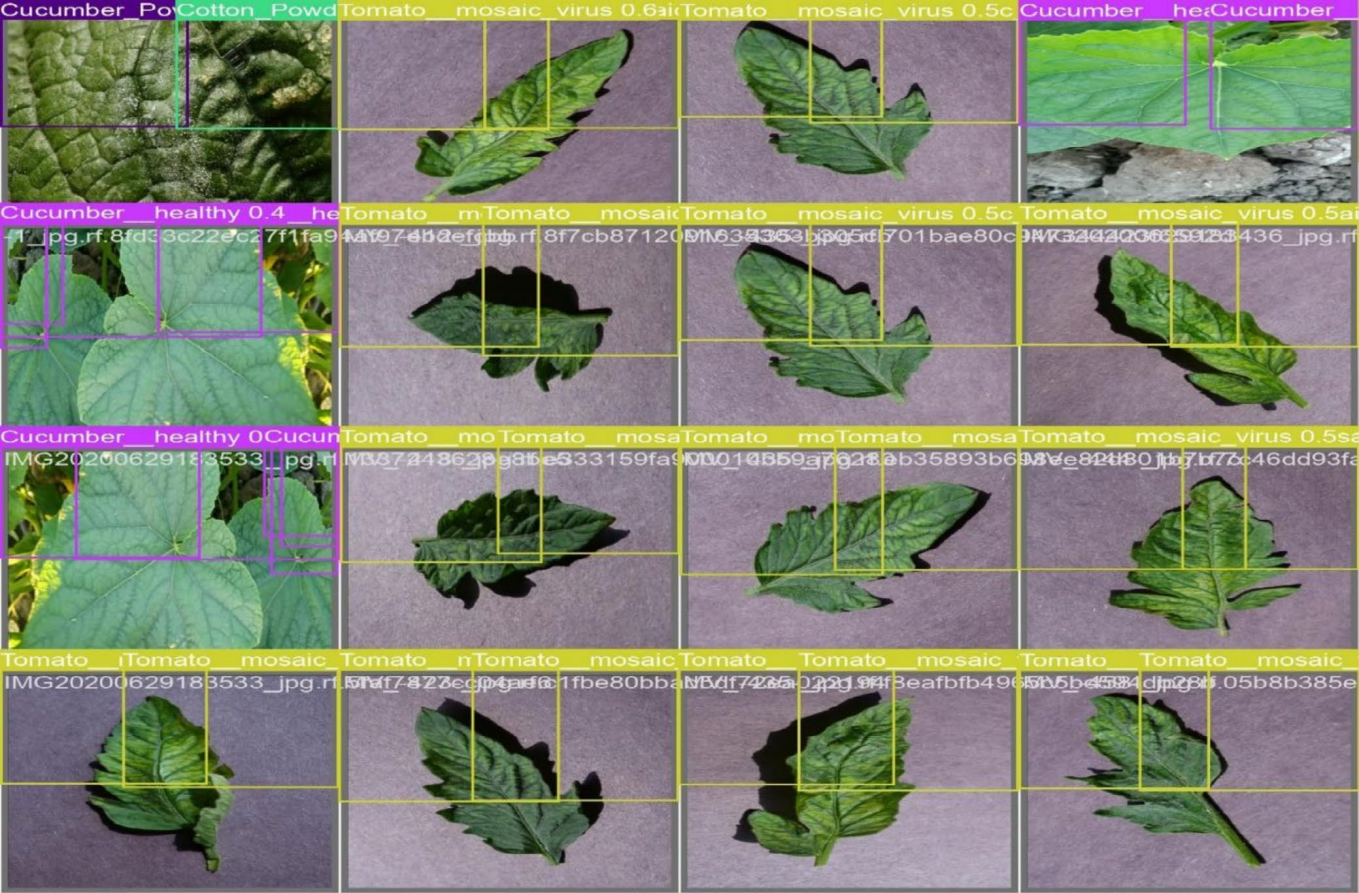
Validating batch 0 predictions.

The model shown in Figure 15 accurately detects and labels crop diseases such as “Tomato_mosaic_virus” and “Cucumber_healthy,” with bounding boxes surrounding the impacted areas. The validation results for batch 0 labels are shown in the first picture. The annotations show how well the model can distinguish between healthy and sick crops. Similarly, the validation of labels is evaluated with the predictions, which shows that in Figure 16, predictions successfully identify and categorize the crop illnesses, demonstrating the model's excellent precision and recall, much as the labeled validation.

**FIGURE 17:**
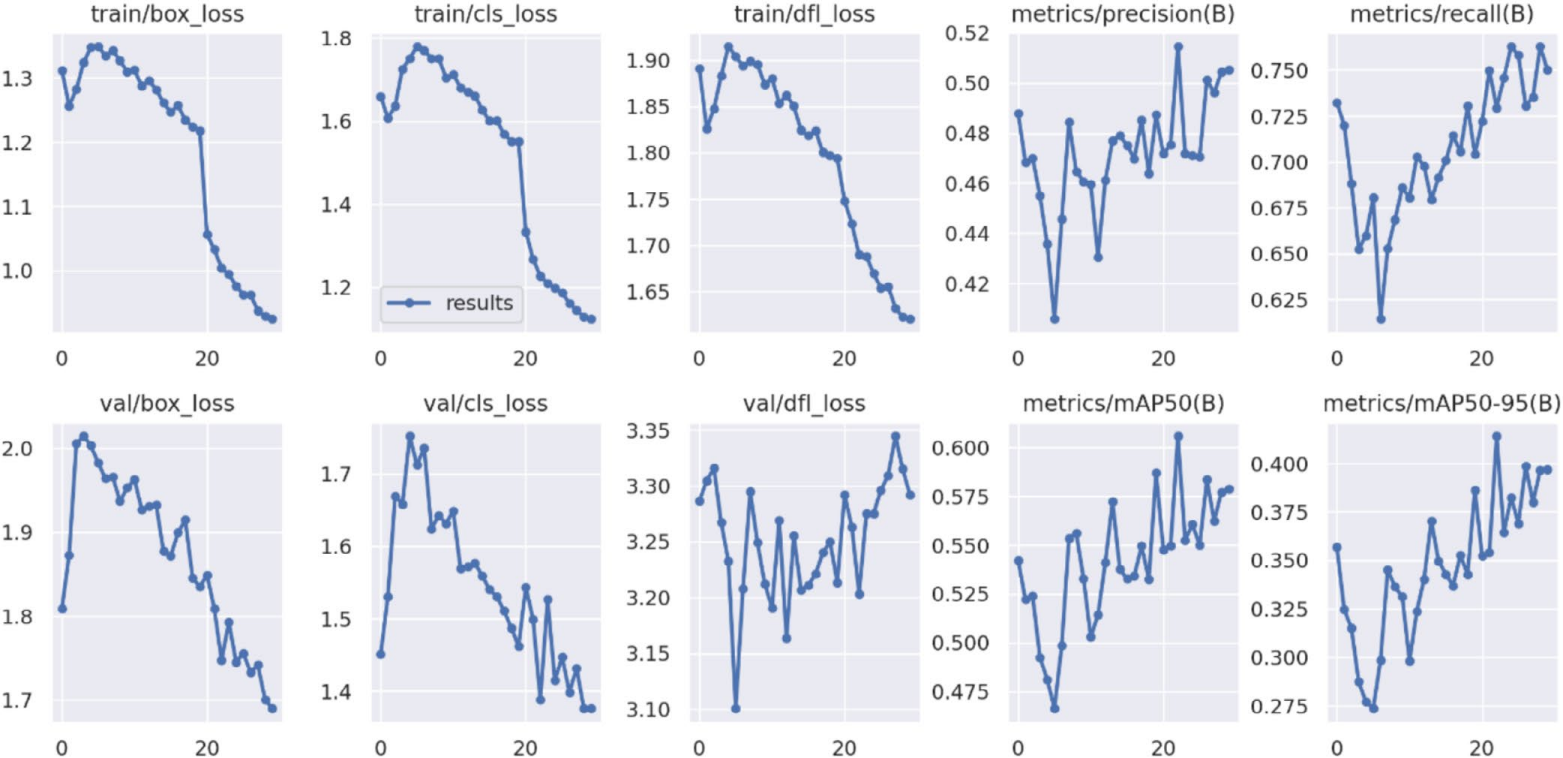
Various detection results.

In Figure 17, the YOLOv8 model's crop disease detection results show notable gains in performance across a range of measures during the training and validation stages. The train/box_loss, train/cls_loss, and train/dfl_loss training loss metrics all exhibit a notable decline: train/box_loss falls from around 1.3 to 0.8, train/cls_loss falls from 1.7 to 1.0, and train/dfl_loss falls from roughly 1.9 to 1.5. Comparably, there are noticeable drops in the validation losses (val/box_loss, val/cls_loss, and val/dfl_loss). Specifically, val/box_loss goes from 2.0 to 1.6, val/cls_loss from 2.0 to 1.5, and val/dfl_loss goes from 3.3 to 3.1.

Metrics of performance, like metrics/precision (B) and metrics/recall (B) for bounding boxes, demonstrate a significant improvement. With fewer false positives and more genuine positives, precision rose from 0.42 to 0.50 and recall from 0.62 to 0.75, respectively, suggesting improved detection capabilities. The mean average precision metrics also showed a considerable improvement, both at the 50% intersection over the union threshold (metrics/mAP50 [B]) and at different thresholds (metrics/mAP50‐95 [B]). A rise in mAP50 from 0.55 to 0.60 and a rise in mAP50‐95 from 0.30 to 0.40 demonstrate the model's high accuracy and robustness in identifying crop diseases.

**FIGURE 18:**
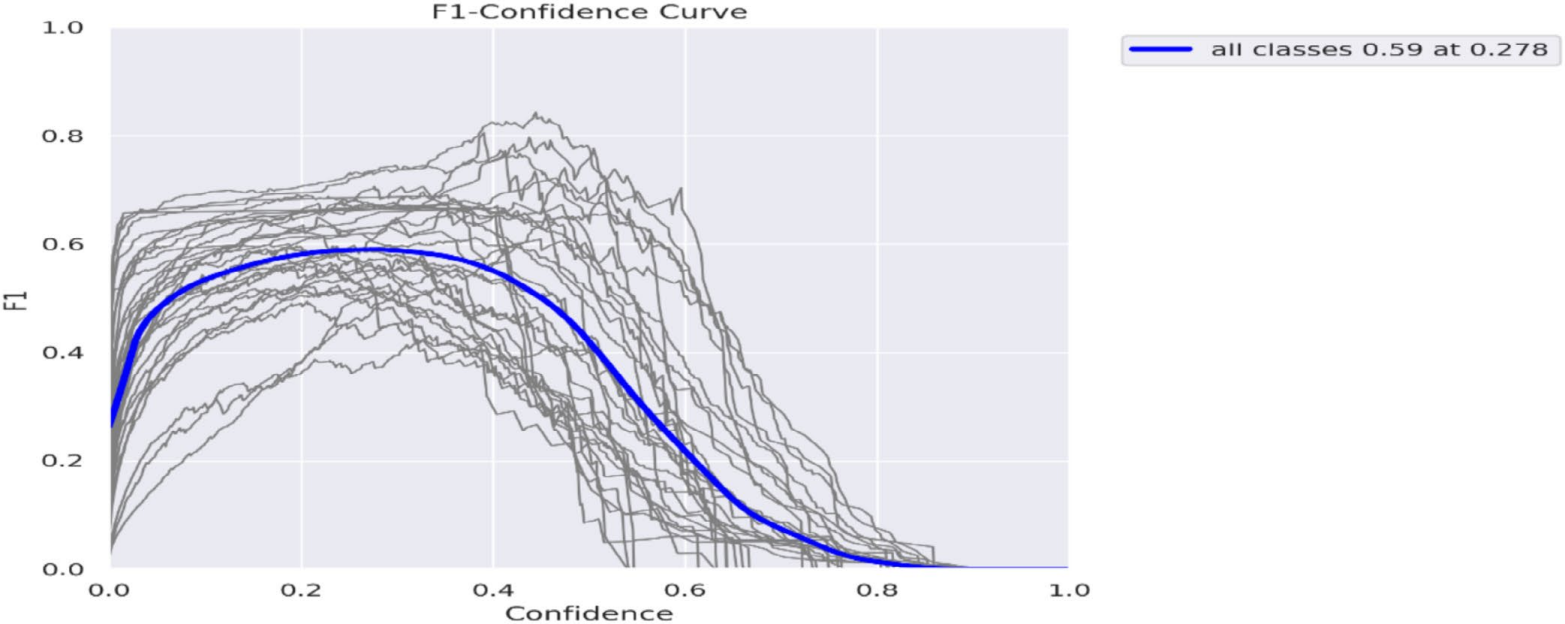
F1‐curve.

In Figure 18, the F1 confidence curve shows that the confidence is depicted, the F1 curve, which peaks at 0.59 when the confidence is approximately 0.278. This curve indicates an ideal balance between precision and recall at this confidence level.

**FIGURE 19:**
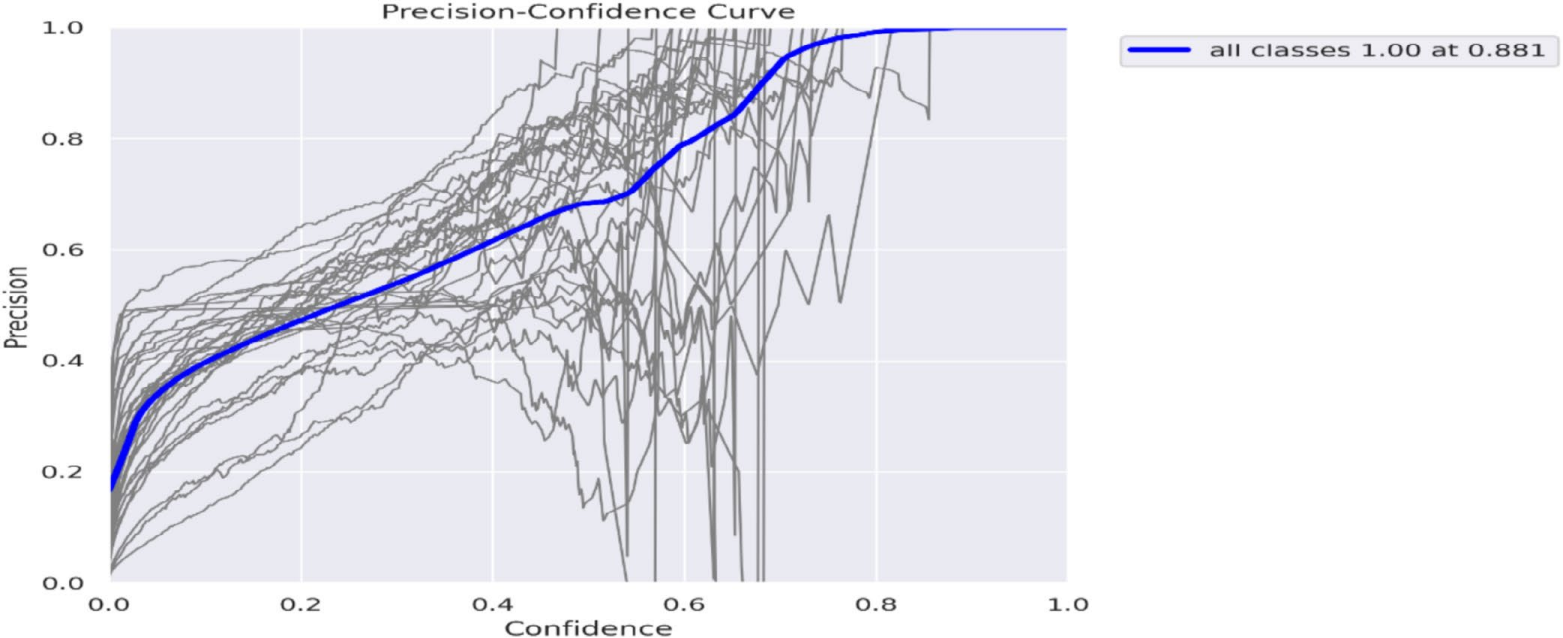
Precision curve.

In Figure 19, the precision curve corresponding to the confidence curve shows how precision increases progressively with confidence until it reaches 1.0 at a confidence level of 0.881.

**FIGURE 20:**
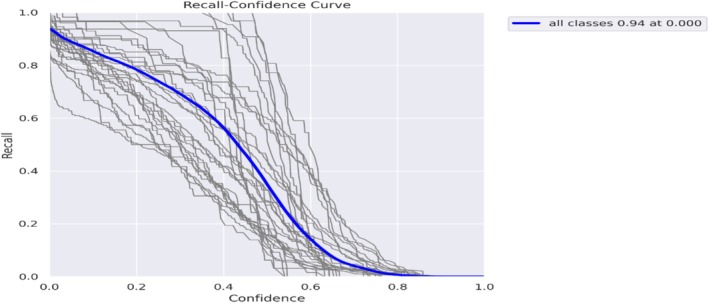
Recall curve.

In Figure 20, the confidence level of 0 shows a falling recollection with increasing confidence, culminating in a high recall of 0.94. Together, these curves shed light on the model's performance and aid in determining the best confidence threshold for striking a balance between recall and precision.

**FIGURE 21:**
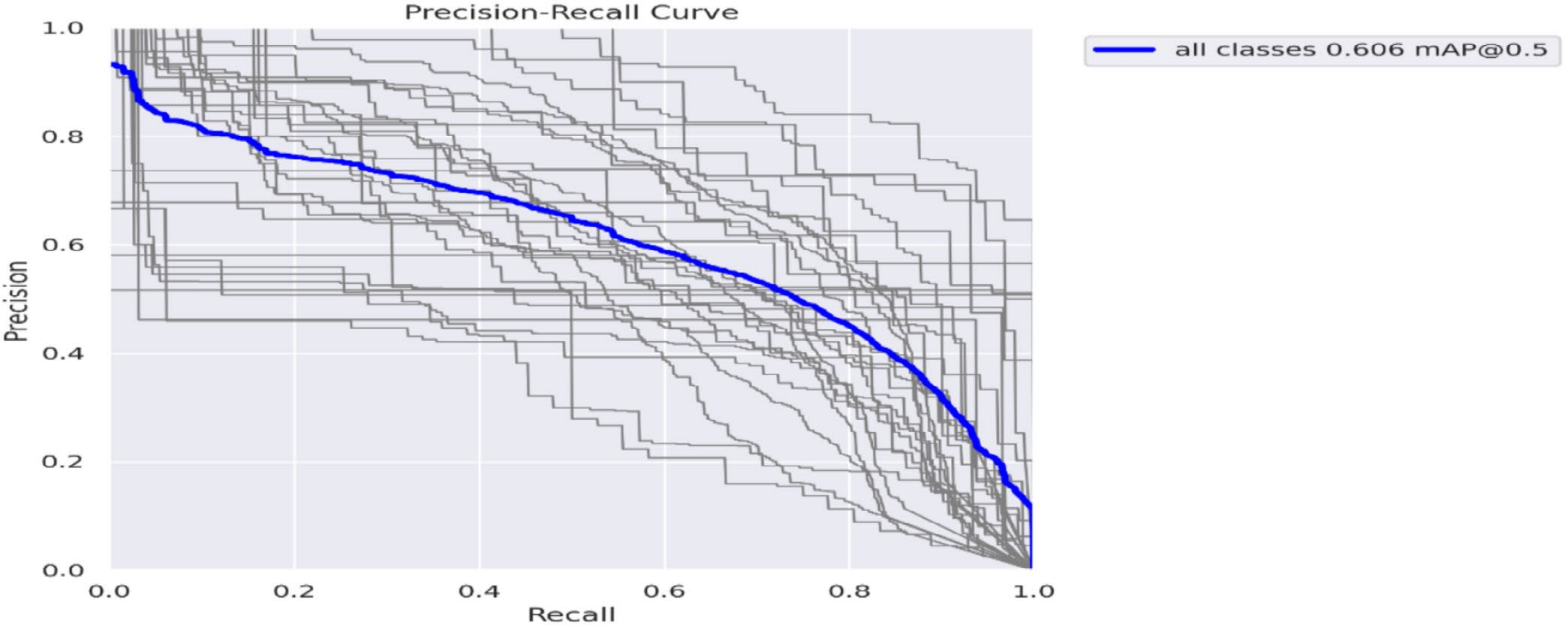
Precision‐recall curve.

Figure 21 shows that at an intersection over the Union (IoU) threshold of 0.5, the blue line in the curve indicates the aggregated performance across all classes, with a mean average precision (mAP) of 0.606. According to this curve, the model keeps a decent balance between recall and precision, with a slight decrease in precision occurring at higher recall values. The gray lines show the differences in detection accuracy between various diseases and represent individual class performances.

**FIGURE 22:**
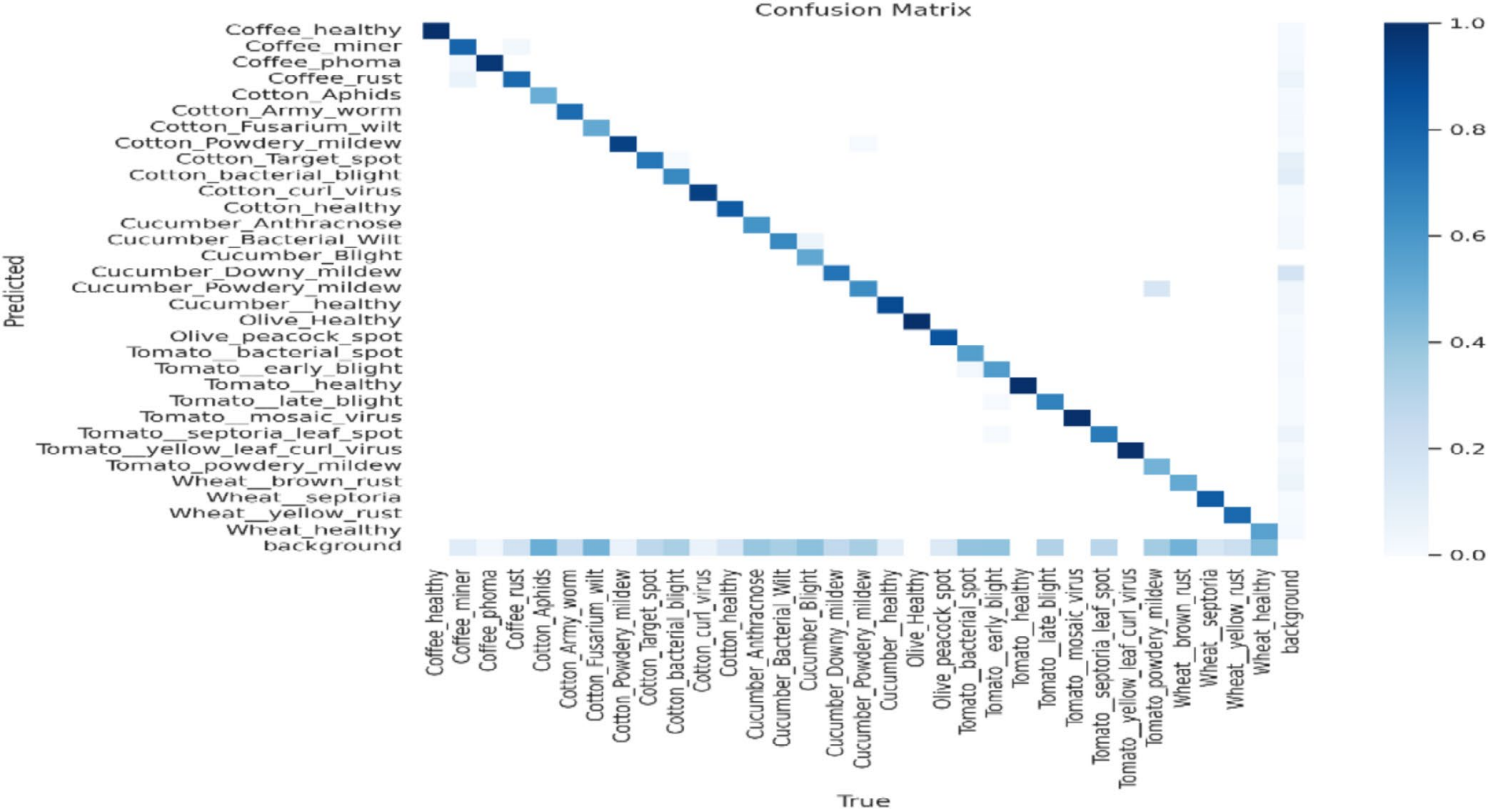
Confusion matrix.

Figure 22 is the confusion matrix of evaluation metrics, which shows the model prediction accuracy for multiple classes. Each cell shows the number of expected occurrences for a given class relative to the actual class. Off‐diagonal cells show misclassifications, while darker diagonal cells show better rates of proper categorization. The matrix indicates that the model can correctly identify most diseases, while there is confusion between similar diseases. Overall, it shows a good result, and the model is robust enough to detect crop diseases well.

### 4.3 Classification

**FIGURE 23:**
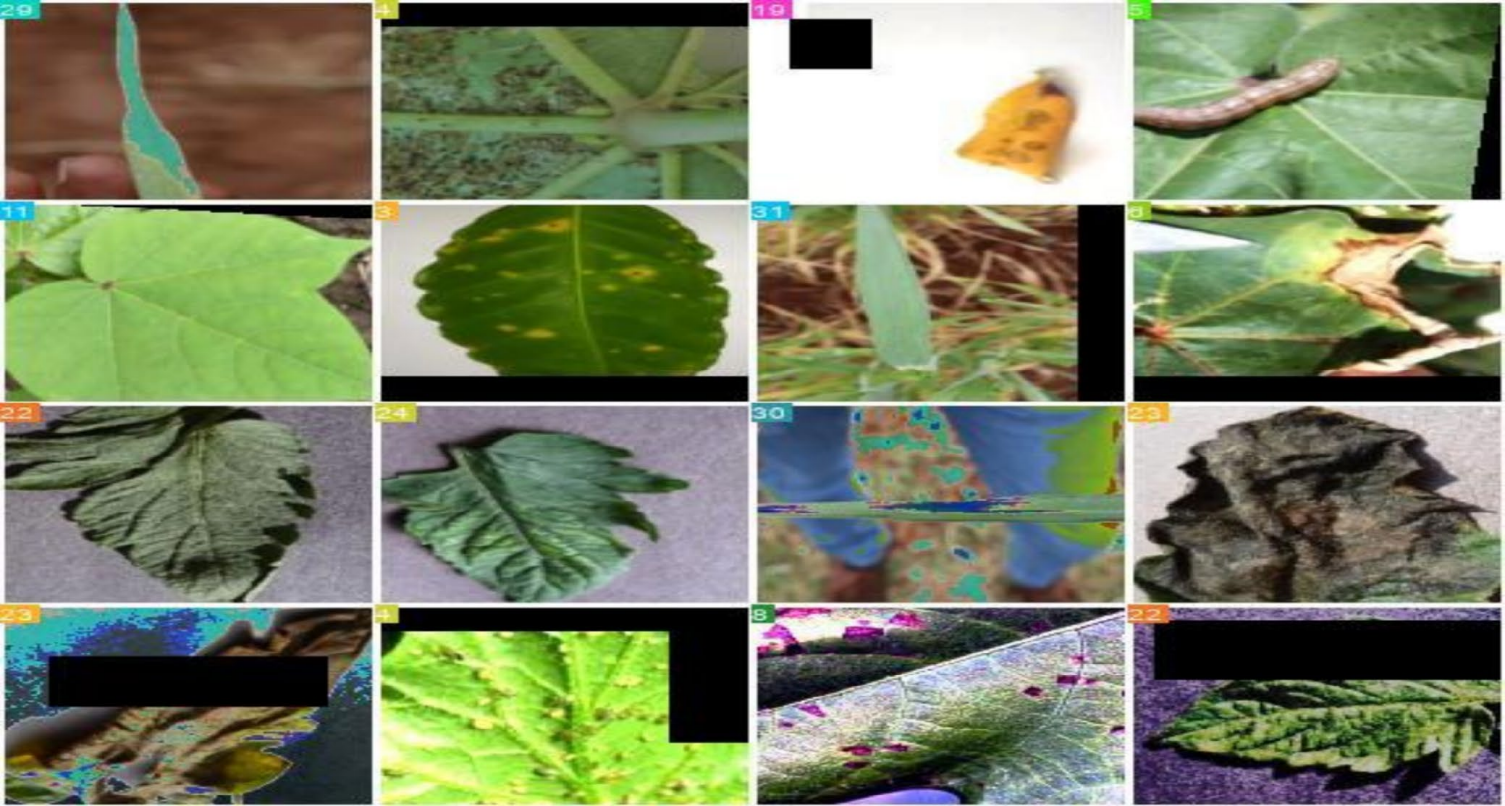
Training Batch 0.

In Figure 23, to make separate image tracking easier during training, each image is labeled with a unique identifier that shows up as a colored number in the image's top‐left corner. The dataset's visual diversity—which includes a range of leaf textures, colors, and disease patterns—ensures thorough learning and helps the model generalize effectively to a wide range of real‐world situations. This batch provides the balanced dataset required for efficient training by including photos of healthy and diseased leaves and apparent symptoms, including leaf spots, discoloration, and pest damage.

**FIGURE 24:**
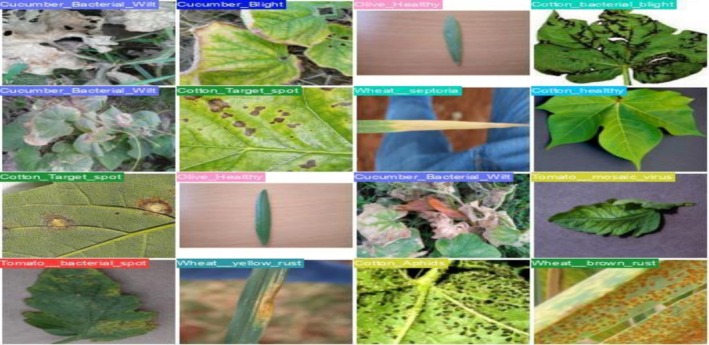
Validation Batch 0 labels.

Figure 24 shows the validation of label 0, which appropriately depicts the crop's real sickness or health state. To ensure that the ground truth of the dataset is dependable, labels like “Cucumber_Bacterial_Wilt,” “Cotton_Target_Spot,” “Wheat Septoria,” and “Olive Healthy” are appropriately applied to the corresponding photos.

**FIGURE 25:**
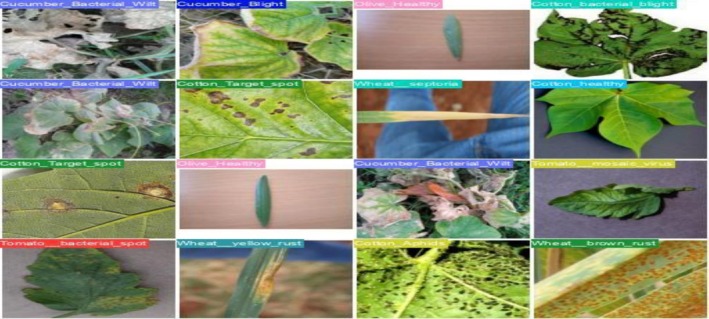
Validation of Batch 0 Predictions.

Figure 25 shows the prediction of the class of a particular crop corresponding to actual labels. The goal is to compare the model's predictions with the ground truth labels to assess the model's accuracy. Any disparities point to areas where the model may need more refining, while successful predictions show that the model has learned to recognize and categorize specific crop diseases.

**FIGURE 26:**
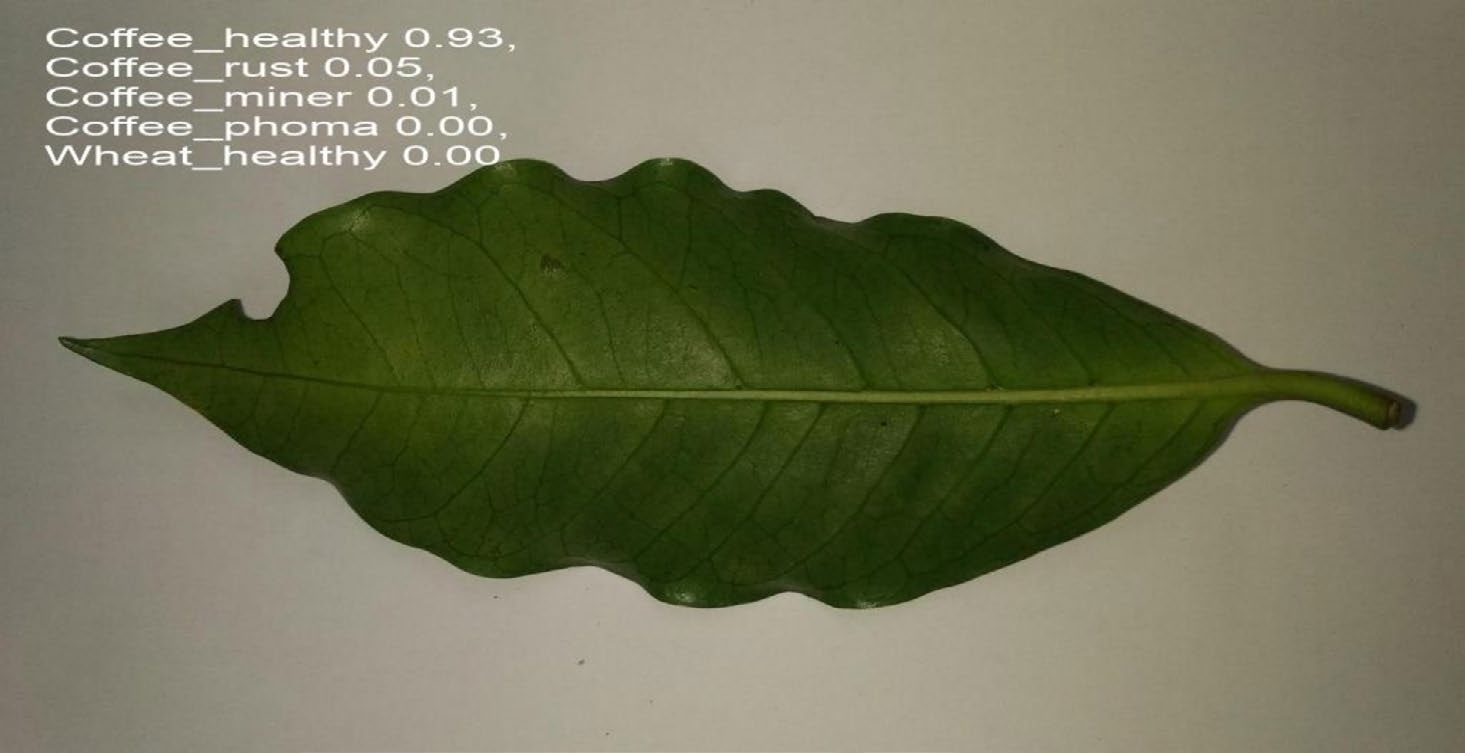
Prediction.

**FIGURE 27:**
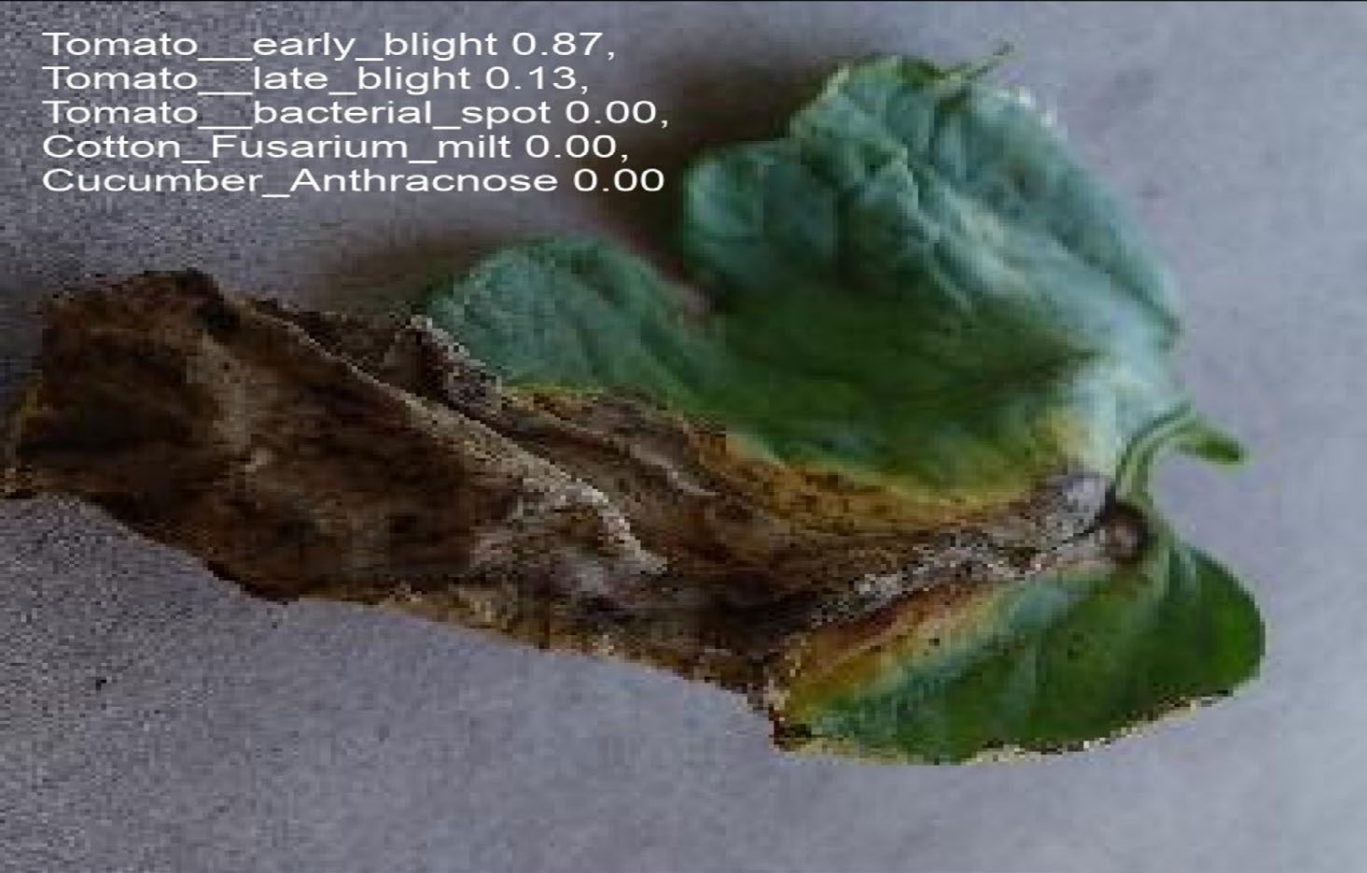
Prediction.

Figures 26 and 27 shows the prediction of a particular disease predicted by the model.

**FIGURE 28:**
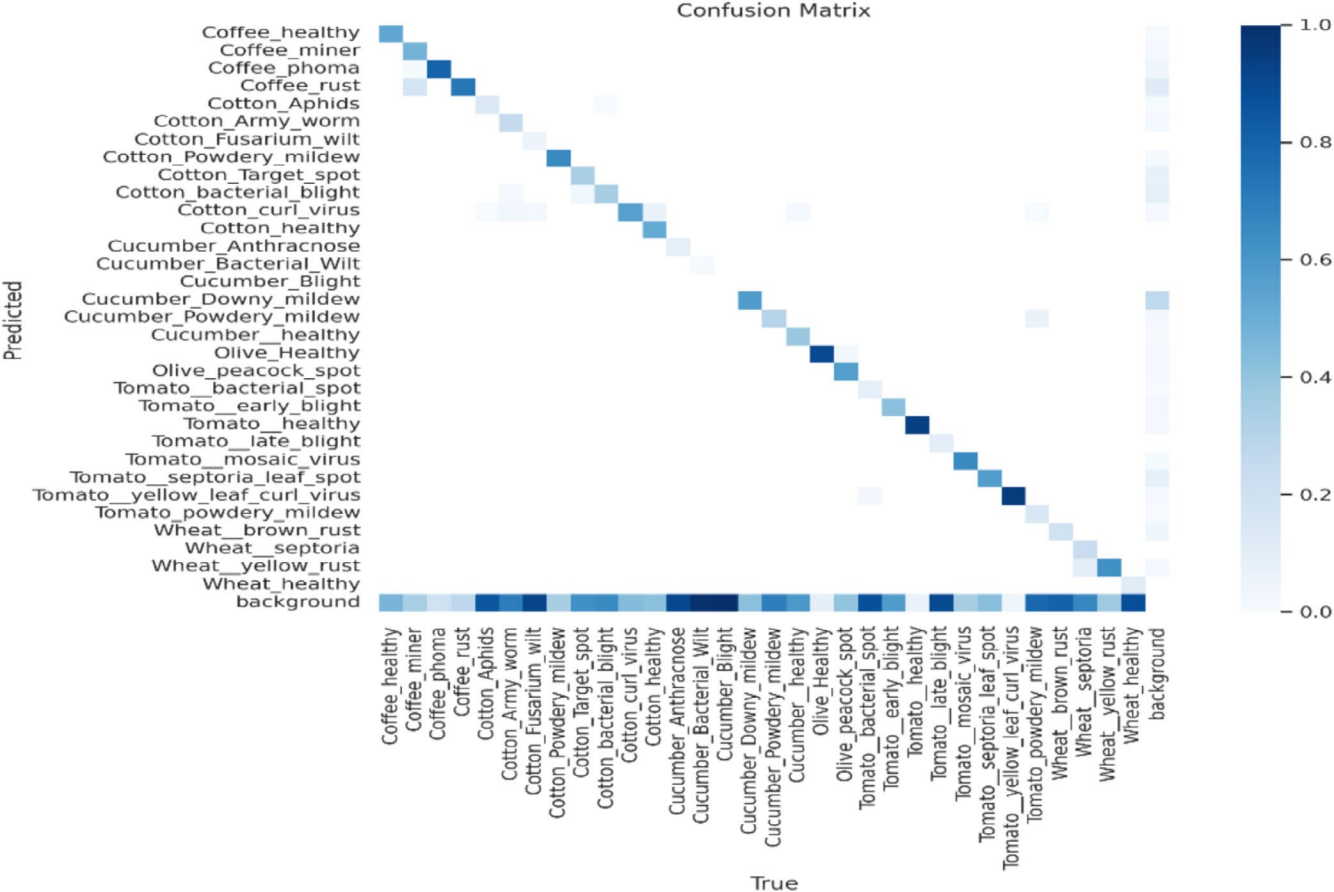
Confusion matrix.

Figure 28 is the confusion matrix of classification, which represents the performance of the classification model and shows the anticipated labels on the vertical axis and the actual labels along the horizontal axis. Every entry in the matrix represents the percentage of cases where a correct label was mistakenly identified as a different one. Each row in the matrix shows the proportion of instances where an accurate label was predicted and mistakenly identified as different. Although there are certain misclassifications, they are also present for specific classes and well‐classified for certain diseases.

The results are compared with the state‐of‐the‐art model, as shown in Table 4.

**TABLE 4:** Comparison with the state‐of‐the‐art models.

References	Model	Accuracy (%)
Nigar et al. (2024)	ResNet‐50	79.83
Jangid and Sharma (2023)	VGG‐16	90
Sardogan et al. (2018)	CNN and Learning Vector quantization	86
Alatawi et al. (2022)	AI‐Based VGG‐16	90.4
Reddy and Adimoolam (2024)	Naïve Bayes	91
Karthik et al. (2024)	SqueezeNet	91.55
Tabbakh and Barpanda (2023)	TLMViT with ResNet‐50	91.74
Thakur et al. (2021)	CNN + ViT	87.87
Our proposed model		92.567

## 5 Conclusion

In this study, we have addressed the crucial problem of detection and diagnosis of crop diseases for the six key crops: olives, coffee, cucumbers, wheat, and tomatoes, and then divided them into 32 different classes. We employed transfer learning with the pre‐trained YOLOv8 model on a hybrid dataset to accomplish segmentation, detection, and classification tasks. This method showed excellent results with a precision of 1.00, a recall of 0.94, and an overall accuracy of 92.567, demonstrating the model's usefulness in recognizing and categorizing a range of agricultural illnesses, significantly outperforming traditional disease detection methods. This work has reduced crop yield losses dramatically through prompt and precise disease diagnosis and detection, and provided the farmers with a timely decision to prevent crops from diseases, which has significant consequences for agricultural productivity. Even with the promising results, our method has a few drawbacks. The model's application in resource‐constrained environments may be limited due to its high computational resource requirements. Furthermore, performance may vary on diverse sets of complex images, necessitating extensive training for more optimized results.

In the future, our model's performance and applicability will be improved in several important areas. The integration of extra data sources, such as hyperspectral and multispectral imaging, which capture the information of images at varying wavelengths, will subsequently provide more in‐depth details regarding plants' health and disease states. This improvement can significantly increase the accuracy and resilience of the model, even though the data we now use comes from publicly available sources. Furthermore, increasing the diversity of the samples in the dataset and incorporating more diseases across crops will be essential to enhance the model's generalizability in different geographical areas. We intend to increase the model's training with more computational resources, which will subsequently be significantly improved by this thorough training, offering even more accurate and dependable disease detection capabilities.

## Author Contributions

Muhammad Amir Khan and Muhammad Nouman Noor performed the original writing part, software, and methodology. Muhammad Masab and Tehseen Mazhar performed rewriting, investigation, design methodology, and conceptualization. Farah Haneef, Muzammil Hussain, and Mateen Yaqoob performed the related work part and managed results and discussions. Tehseen Mazhar, Ghadah Aldehim, and Muhammad Amir Khan performed related work part and managed results and discussion. Muhammad Masab, Tehseen Mazhar, and Muhammad Amir Khan performed rewriting, design methodology, and visualization. Farah Haneef and Muzammil Hussain performed rewriting, design methodology, and visualization.

## Funding

This work was supported by Princess Nourah bint Abdulrahman University Researchers Supporting Project number (PNURSP2026R387) and Princess Nourah bint Abdulrahman University, Riyadh, Saudi Arabia.

## Ethics Statement

The authors have nothing to report.

## Conflicts of Interest

The authors declare no conflicts of interest.

## Data Availability

The data used to support the findings of this study are available from the corresponding authors upon request.
